# Effect of Ozone, Clothing, Temperature, and Humidity on the Total OH Reactivity Emitted from Humans

**DOI:** 10.1021/acs.est.1c01831

**Published:** 2021-09-30

**Authors:** Nora Zannoni, Mengze Li, Nijing Wang, Lisa Ernle, Gabriel Bekö, Pawel Wargocki, Sarka Langer, Charles J. Weschler, Glenn Morrison, Jonathan Williams

**Affiliations:** † Atmospheric Chemistry Department, Max Planck Institute for Chemistry, 55128 Mainz, Germany; ‡ International Centre for Indoor Environment and Energy, Department of Civil Engineering, Technical University of Denmark, 2800 Lyngby, Denmark; § IVL Swedish Environmental Research Institute, 41133 Göteborg, Sweden; ∥ Division of Building Services Engineering, Department of Architecture and Civil Engineering, Chalmers University of Technology, 41296 Göteborg, Sweden; ⊥ Environmental and Occupational Health Sciences Institute, Rutgers University, Piscataway, New Jersey 08854, United States; # Department of Environmental Sciences and Engineering, Gillings School of Global Public Health, The University of North Carolina at Chapel Hill, Chapel Hill, North Carolina 27599-7431, United States

**Keywords:** indoor chemistry, volatile organic compounds, human skin emissions, indoor ozone, ozone deposition velocity

## Abstract

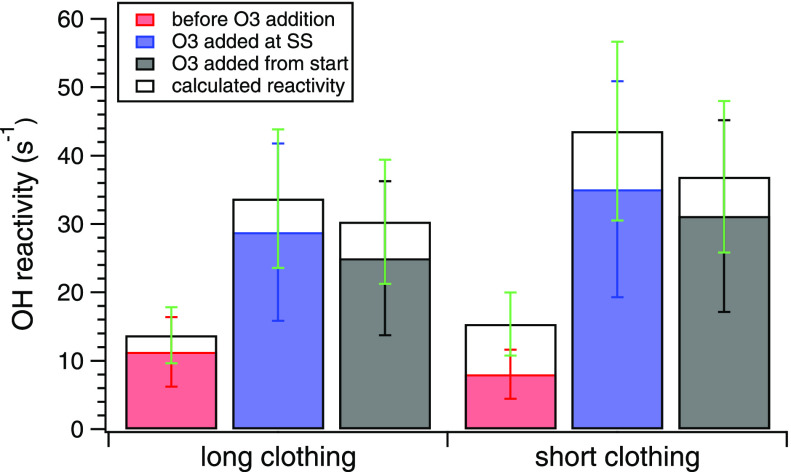

People influence indoor air chemistry through their chemical emissions via breath and skin. Previous studies showed that direct measurement of total OH reactivity of human emissions matched that calculated from parallel measurements of volatile organic compounds (VOCs) from breath, skin, and the whole body. In this study, we determined, with direct measurements from two independent groups of four adult volunteers, the effect of indoor temperature and humidity, clothing coverage (amount of exposed skin), and indoor ozone concentration on the total OH reactivity of gaseous human emissions. The results show that the measured concentrations of VOCs and ammonia adequately account for the measured total OH reactivity. The total OH reactivity of human emissions was primarily affected by ozone reactions with organic skin-oil constituents and increased with exposed skin surface, higher temperature, and higher humidity. Humans emitted a comparable total mixing ratio of VOCs and ammonia at elevated temperature-low humidity and elevated temperature-high humidity, with relatively low diversity in chemical classes. In contrast, the total OH reactivity increased with higher temperature and higher humidity, with a larger diversity in chemical classes compared to the total mixing ratio. Ozone present, carbonyl compounds were the dominant reactive compounds in all of the reported conditions.

## Abstract 2

In the presence of ozone, humans emit more OH-reactive compounds at higher indoor temperatures and humidity, and with less clothing coverage.

## Introduction

On average, we spend 85–90% of our time indoors,^1,2^ that is, at home, in offices, and schools. While indoor environments afford some protection against outdoor ozone and fine particles, confined volume and limited ventilation can increase exposure to multiple volatile organic and inorganic compounds from indoor sources.^3^ Indoor air chemistry is influenced by the strength and nature of the indoor sources, the ventilation rate and quality of outdoor air, the ratio of exposed surfaces to volume of the space, and occurrence of natural light sources that drive photochemistry outdoors.^4^ In occupied environments, human beings and their associated activities such as cooking and cleaning represent the dominant source of gaseous and particulate emissions.^5,6^ Human beings emit numerous reactive volatile organic compounds (VOCs) through their breath and skin.^7−10^ The main VOCs reported from breath emissions of healthy individuals include hydrocarbons such as isoprene, alcohols, ketones, and compounds containing nitrogen and sulfur.^11^ Common skin VOCs include compounds directly emitted by the body as well as those resulting from oxidation of such compounds (e.g., 6-methyl-5-hepten-2-one and geranyl acetone). In total, approximately 500 compounds have been identified as emissions from skin, compared to almost 900 identified in breath.^7,12^ Ozone (O_3_), the main indoor oxidant, is also the main chemical sink of human emissions, although present at only 10–70% of the outdoor levels due to losses (reaction) during the building envelope penetration and on indoor surfaces.^13^ Ozone deposition velocities have been calculated for materials used in building furnishings,^14−16^ occupied environments,^8,17^ aircrafts interiors,^18^ skin,^19^ hair,^20^ and clothing.^18,19,21^ Values can range from <1 to >20 m/h,^22^ but the central tendency for area-averaged surfaces indoors is about 1.4 m/h. The largest reported deposition velocities were associated with human skin, soiled hair, and clothing. Adult human skin has a large surface area (1.5–2.0 m^2^) and, due to secretions from glands across the body, it is covered with proteins and lipids such as fatty acids, triglycerides, cholesterol, and squalene (C_30_H_50_).^23^ The latter contributes roughly to 50% of the C=C bonds available for O_3_ reactions.^20^ Squalene reacts rapidly with O_3_ generating first-generation products including ketones such as acetone (C_3_H_6_O), 6-methyl-5-hepten-2-one (6-MHO, C_8_H_14_O), and geranyl acetone (C_13_H_22_O). Further O_3_ reactions with the primary products lead to secondary products such as additional acetone, 4-oxopentanal (4-OPA, C_5_H_8_O_2_), 1,4-butanedial (C_4_H_6_O_2_), and levulinic acid (C_5_H_8_O_3_). The generation of gas-phase products is dependent on environmental factors such as O_3_ concentration and relative humidity.^24^

One of the most critical aspects for understanding indoor air chemistry and quality is a comprehensive knowledge of the VOCs present indoors and emitted by humans,^25^ as well as their reactivity and lifetime. A relatively new measurement, namely, the total hydroxyl radical (OH) reactivity,^26^ allows direct assessment of the entire chemical budget of compounds that react with the OH radical. Coupled with measurements of individual VOCs, it can be used to determine how complete is the chemical characterization of reactive organic compounds in air. The total OH reactivity is defined as the total loss frequency of OH, the inverse being the OH lifetime. Since the development of the concept of total OH reactivity as a “top-down” parameter,^26^ a number of instruments measuring OH reactivity have been deployed in a diverse set of outdoor environments,^27−30^ with the goal of investigating budgets of reactive VOCs as well as OH^31^ and O_3_.^32^

The Indoor Chemical Human Emissions and Reactivity (ICHEAR) project aimed at quantifying VOC emissions and OH reactivity from adult volunteers exposed in groups of four in a controlled climate chamber.^33^ In Wang et al.,^34^ we analyzed the OH reactivity of isolated breath and dermal emissions as well as whole-body emissions from volunteers of different age groups in the presence and absence of O_3_. We showed that the reactive VOCs budget was closed, that when O_3_ was present, “OH reactivity approximately doubled” with the increase due chiefly to unsaturated secondary products of O_3_-skin-oil chemistry (57%), and that no significant difference was observed among different age groups.^34^ In this study, using results from a different set of experiments, we examine the effects of indoor O_3_ concentration, clothing, temperature, and relative humidity on the total OH reactivity of whole body emissions from experiments involving two different groups of adult volunteers.

## Methods

### Experimental Design and Methods

The experiments were conducted in a 22.5 m^3^ stainless steel climate chambers at the Technical University of Denmark (DTU) as part of the ICHEAR project (Table S1). The focus of this research was on human bioeffluents, so other emission sources were minimized. The chamber was furnished with a table and four wire-meshed chairs, and measurements of the empty chambers were taken before volunteers entered to characterize background conditions. Prefiltered outdoor O_3_-free air was used to ventilate the chamber with an average air change rate (ACR) of 3.2 h^–1^. Mixing was ensured with two fans in the chamber, directed to the walls. The environmental conditions were either moderate temperature and low humidity (set points 25 °C and 25%, respectively), or high temperature and high humidity (set points 31 °C and 65%, respectively). Ozone was added into the supply air either before the start of the experiment, so that its concentration had reached steady state (SS) before the volunteers entered (short experimental days—morning only), or it was introduced into the chamber air after the human emitted volatile organic compounds reached steady state (long experimental days—morning and afternoon). Although the two scenarios result in identical steady-state ozone concentration with occupancy (around 35 ppb), they have different dynamics; when steady state is approached from an ozone-rich state more ozone-initiated chemistry occurs than when steady state is approached from an ozone-poor state.^35^ In the latter case, the volunteers left the chamber for a 10 min lunch break after 3 h of O_3_-free condition. Ozone generation commenced 10 min after they returned to the chamber for another 2.5 h exposure. The target O_3_ mixing ratio in the unoccupied chamber was ∼100 ppb, which resulted in a chamber level of ∼35 ppb when four volunteers were present.

Two groups with young adult volunteers participated in the present experiments, originally termed as groups A1 and A2.^31^ Each group was composed of two females and two males being ∼25 years old. They were asked to refrain from drinking alcohol and eating spicy food one day prior to and during the days of the experiments. The night before each experimental day they were washed with provided fragrance-free soap and shampoo; no shower was taken in the morning. During the experiments, they wore a provided set of new prewashed (fragrance-free detergent) and tumble-dried “long” or “short” clothing. “Long clothing” consisted of sweatpants, long-sleeve shirts, and calf socks, while “short clothing” consisted of shorts, t-shirts, and ankle socks.

Separate experiments on O_3_ exposure (concentration at the inlet ∼100 ppb, ACR ∼3.2 h^–1^) of clothing only were conducted with four clean t-shirts and four soiled (worn) t-shirts, of the same type given to the volunteers. Soiled t-shirts were worn overnight and hung inside-out in the chamber. Detailed information on the experimental design is provided in Bekö et al.^33^

Total OH reactivity was measured using a custom-built comparative reactivity method (CRM) instrument previously deployed in multiple outdoor atmosphere measurement campaigns^32^ and validated in a large inter-comparison exercise.^36^ The CRM^37^ combines a glass flow reactor with a proton transfer reaction-quadrupole mass spectrometer (PTR-QMS, Ionicon Analytik GmbH, Austria^38^) to measure the concentration of a reference compound normally not found in ambient or indoor air (here pyrrole, C_4_H_5_N), whose reaction rate with OH is known, and whose concentration can be unambiguously detected by PTR-MS (C_4_H_5_NH^+^, *m*/*z* 68). The PTR-MS was operated at standard conditions (*P*_drift_ = 2.2 mbar, E/N = 130Td, *T*_inlet_ = 60 °C) to monitor *m*/*z* 68 with a dwell time of 20 s. A pressurized gas standard of pyrrole (Westfalen AG, Germany) was diluted in the flow reactor and the concentration, *C*, was measured under three sequential conditions: with clean air and dry N_2_ after photolysis (*C*1), after OH is generated (*C*2), with chamber air replacing clean air (*C*3). Hydroxyl radicals were generated in the glass flow reactor through photolysis of water vapor (Hg UV lamp emitting at 184.9 nm). Assuming a pseudo-first-order kinetics regime inside the reactor ([pyrrole] ≫ [OH]), the OH reactivity *R*_air_ was determined from pyrrole concentrations *C*1, *C*2, and *C*3 with eq 1
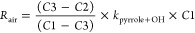
1where *k*_pyrrole+OH_ is the rate constant of the reaction between pyrrole and OH and equals (1.20 ± 0.16) × 10^–10^ cm^3^ molecule^–1^ s^–1^.^39,40^ The concentration *C*1 was quantified after each experiment using an OH scavenger.^41^ The OH scavenger used during the campaign was propane at a concentration ∼3.6 × 10^3^ ppm inside the reactor. The pyrrole mixing ratio *C*1 measured when the reactor was wet with the OH scavenger was 59 ± 15 ppb (mean campaign value ±1σ), and the pyrrole mixing ratio *C*1 measured when the reactor was dry without any OH scavenger was 55 ppb. An automated system switched between clean air (to determine *C*2) and chamber air (to determine *C*3) every 5 min. The data processing consists of a PTR-MS calibration with pyrrole at different levels of humidity, humidity correction on *C*2 to correct for OH recycling when humidity changed, NO*_x_* and O_3_ corrections on *C*3 for OH recycling, reactivity calibration for deviation from pseudo-first-order kinetics with test gas having different OH rate coefficients, and dilution of the sampling flow into the flow reactor. All correction factors were determined experimentally.^34,42^ Correction for NO*_x_* and O_3_ interference for recycling OH were not necessary as the NO concentration measured inside the chamber was ∼1 ppb (corresponding to a change in reactivity <3%), and increasing concentrations of O_3_ (0–110 ppb) were tested and found not to interfere with the chamber CRM set-up. The resolution of the OH reactivity measurements was 1–10 min, the limit of detection (1σ) was ∼5 s^–1^ and the quantified total uncertainty was ∼48%. The total uncertainty was calculated as the propagated uncertainty on the pyrrole standard (10%), on the rate constant of the reaction between pyrrole and OH (14%), on the dilution of the sampled flow (0.16%), on the corrections used (31% for kinetics conditions, 29% for humidity), and on signal precision (∼15%).

Ancillary measurements performed during the ICHEAR campaign and used in this study included: volatile organic compounds (VOCs) detected with a PTR-MS equipped with a time of flight detector (ToF), and with a fast gas chromatograph–mass spectrometer (fast GC-MS); carbon dioxide (CO_2_) and ammonia (NH_3_) detected with cavity ring-down spectroscopy (Picarro, G2401 and G2103, respectively), ozone (O_3_), temperature and relative humidity (RH).^33,34,43^

A common fluorinated ethylene propylene (FEP) inlet (OD 1/2 in., length 5 m) was installed in the chamber’s air exhaust outlet and air was drawn (sampling flow ∼7 L min^–1^) to the sampling devices to measure VOCs, OH reactivity, and CO_2_ concentration. Background measurements of the supply air just before it entered the chamber were taken periodically before, during, and after each experiment. Ozone was measured from a separate inlet in the middle of the chamber, and the air temperature and humidity sensors were placed in the chamber.

PTR-ToF-MS (PTR-ToF-MS 8000, Ionicon Analytik GmbH, Austria^38^) was operated under standard conditions (*P*_drift_ = 2.2 mbar, *T*_inlet_ = 60 °C and E/N = 137Td) with *m*/*z* up to 450 (mass resolution 4000 at 96 amu). A certified gas standard mixture (Apel-Riemer Environmental Inc.) containing 14 compounds was used to calibrate the instrument during the campaign. Calibrations were performed at stepwise increasing concentrations at different humidity levels (10–80%). The sensitivity was generally not affected by humidity (below 10% for most compounds, except for benzene and toluene, where sensitivity varied within 20%). A humidity-dependent sensitivity curve was derived and used to determine the concentrations of the species included in the calibration gas. A theoretical method was applied to determine the mixing ratios of the masses of the compounds not included in the gas standard.^44^ The time resolution of the measurements was 20 s, the uncertainty was up to 11% for the compounds in the calibration mixture (including uncertainty derived from the humidity dependency), and ∼50% for those compounds not included in the calibration mixture.

The fast GC-MS instrument (SOFIA—System for Organic Fast Identification Analysis^45^) was equipped with a cryogenic pre-concentrator. A sampling volume of 20–40 mL was collected. A quartz particle filter impregnated with a 10% w/w solution of sodium thiosulfate was installed at the inlet to scrub O_3_. Calibrations were performed using a certified gas standard mixture (Apel-Riemer Environmental Inc.) containing 79 compounds. The time resolution of the measurements was 3 min, the limit of detection was <25 ppt, and the uncertainty was <10%.

The time resolution of CO_2_ and NH_3_ was 2 s. Ozone was monitored with a 2B Technologies model 205 ozone monitor (2B Technologies, Boulder, CO) with a time resolution of 10 s (accuracy: 1.0 ppb or 2% of reading). Air temperature and RH were monitored with Vaisala GMW90 (accuracy: temperature ±0.5 °C, RH ±2.5% below 60%; Vaisala Corporation, Helsinki, Finland) connected to a HOBO UX120-006M 4-channel analog data logger (Onset Computer Corporation, Bourne, MA) with a time resolution of 1 min.^33^

### Skin Wipes Samples

Skin wipes were collected from the volunteers’ right forearm before and from the left forearm after each experiment. Pre-cleaned sterilized cotton pads wetted with 2-propanol were used to thoroughly wipe (7 strokes) a skin area of 100 cm^2^. Squalene (C_30_H_50_) and pyroglutamic acid (C_5_H_7_NO_3_) concentrations were determined in the collected samples through extraction with an organic solvent and analyzed by gas and liquid chromatography coupled with mass spectrometry GC/MS and LC/MS/MS.

### OH Reactivity Budget

Volatile organic compounds and ammonia concentrations (*X*_*i*_) and their rate coefficients of reaction with OH (*k*_*i*+OH_) were used to determine the species-specific OH reactivity. The sum of species-specific OH reactivities for all measured compounds yields the summed calculated OH reactivity (eq 2)
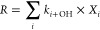
2Table S2 lists the compounds used to calculate the reactivity. From the measured PTR-ToF-MS masses (21–450 *m*/*z*), only masses above the instrumental limit of detection and known to be related to human emissions (higher mixing ratios detected from the occupied chamber in comparison with the unoccupied chamber) were selected to calculate their OH reactivity. The uncertainty of the calculated OH reactivity (∼30%) was determined from the propagation of the uncertainty of the measured concentration *X*_*i*_ (10–50%) and the error in the rate coefficient of the reaction between *i* and OH (*k*_*i*+OH_, 10–100%). The comparison between the total measured OH reactivity and the calculated OH reactivity allows verification of whether all of the OH-reactive VOCs were identified and quantified. As discussed in Wang et al.,^34^ the length and material of the sampling system used during the ICHEAR campaign differed from those used in previous experiments,^8,35^ and this may have led to sampling artifacts. Specifically, in the presence of ozone, adsorption of geranyl acetone on the main inlet FEP surface may have led to consequent reactions with ozone, decreasing the measured steady-state concentration of geranyl acetone and increasing that of 6-MHO. This artifact would not affect the VOC budget analysis reported in this work as the OH reactivity and VOCs were measured with the same sampling system.

### Ozone Deposition Velocities on Occupant Surfaces

The first-order rate constant for O_3_ loss by all mechanisms, *k*_d_, was calculated based on eq 3(6)
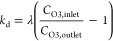
3where *C*_O3,inlet_ is the chamber inlet O_3_ mixing ratio, *C*_O3,outlet_ is the chamber outlet O_3_ mixing ratio, and λ is the air change rate. For an occupied chamber, O_3_ loss is dominated by deposition to the occupants; in this case, the loss rate constant in eq 3 is equal to that associated with the occupant, *k*_occ_. For experiments in which only clean or soiled t-shirts are present in the chamber, O_3_ loss is dominated by deposition to clothing and the removal rate constant is *k*_cloth,only_. Ozone removal associated with chamber surfaces was negligible compared to that on occupants or clothing surfaces (*k*_chamber_ = 0.15–0.17 h^–1 ^^33,35^). For surface removal, each rate constant can be expressed as the product of a mass transfer coefficient (deposition velocity, *v*) and the area of the reactive surface, *A*, divided by the chamber volume, *V*. Therefore
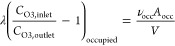
4where the subscripts refer to occupants (occ). The deposition velocity to the four occupants, *ν*_occ_, can be solved directly from eq 4 using the total body surface area (BSA) of the participants calculated according to Du Bois and Du Bois^46^ (7.3 m^2^ in agreement with the average BSA 1.9 m^2^ of a healthy adult^47^). For an experiment with clothing only (no occupants) in the chamber, *v*_cloth,only_ can be solved directly from eq 5, where *A*_cloth,only_ is 0.7 m^2^ per medium-sized shirt times the number of shirts deployed in the chamber.
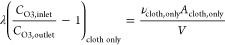
5

### Reactivity-Influencing Factors

Dominance analysis is designed to determine the relative importance of one variable over another in a given data set.^48,49^ It measures the relative importance in all possible subset models in a pairwise manner (in total 2*^n^* – 1 models, *n*: number of variables), i.e., all pairs of variables are compared.^50^ The dominance of one variable over another is calculated by comparing their incremental *R*^2^ contributions over the complete data set.^48,49^ In this study, dominance analysis (in Python) was applied to determine the relative importance of a set of target variables discussed in the manuscript for the total OH reactivity and the OH reactivity of some important reactive VOCs associated with dermal emissions. Specifically, the following target variables were tested: indoor O_3_ concentration, indoor air temperature and relative humidity, clothing, age, and volunteer group (six variables). Ozone, temperature, RH, and clothing factors are discussed in this manuscript, while age and volunteer groups were discussed in Wang et al.^34^ The clothing factor was used to distinguish between volunteers wearing long clothing or short clothing. As the age factor, the average age of the volunteers occupying the chamber was used. The volunteer group factor was used to distinguish between the five different groups of subjects occupying the chamber during the ICHEAR experiments. Steady-state values of OH reactivity, O_3_, temperature, and RH data were used in these analyses. The data set comprised all experiments performed during the ICHEAR campaign on whole body emissions. For long experimental days, both morning and afternoon steady-state values were used separately in the analyses (*n* = 33).^33^

## Results and Discussion

### Ozone and Clothing Effect

**Figure 1:**
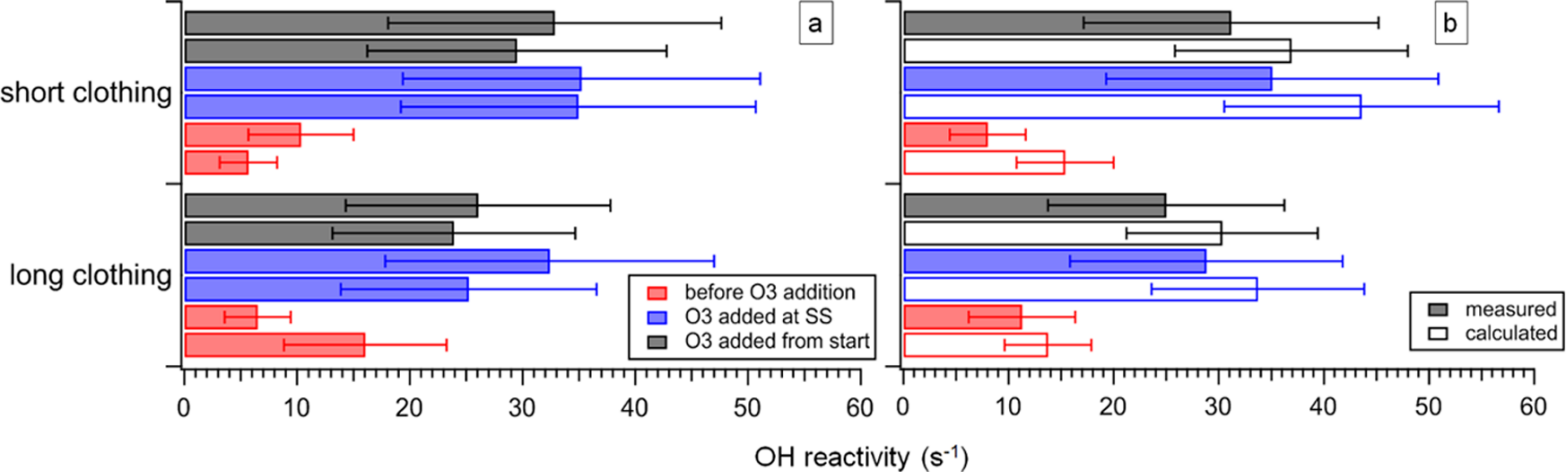
(a) Measured OH reactivity from occupant emissions at steady state. Experiments involved the same four adult volunteers occupying a chamber wearing long clothing or short clothing, under the same conditions on different days. Two replicates were done for the same condition with the same volunteers on different days (*n* = 2). Ozone was absent (red), introduced to the chamber when VOC reached SS (blue), or introduced to the chamber from the start of the experiment (black). (b) Measured (filled bars) and calculated (empty bars) OH reactivity from occupant emissions at steady state. Each bar corresponds to the mean among SS values between the two replicates conducted for each condition. Error bars indicate the method uncertainties (∼48% for measured reactivity, ∼30% for calculated reactivity). Steady-state values were determined during the last 15 min before occupants left the chamber.

Experiments (6), (7), (8), (9) and their respective replicates (21), (22), (23), (24) (Table S1), all conducted with volunteer group A2, probed differences in OH reactivity from occupant emissions under different clothing conditions. During these experiments, the chamber air temperature was moderate (set point 25 °C) and its relative humidity low (set point 25%). Figure 1 shows the measured total OH reactivity (filled bars) and the summed calculated OH reactivity (empty bars) averaged over the final 15 min of each experiment (emissions at steady state^33^) for each condition: long clothing before O_3_ addition (6 and 21 morning); long clothing with O_3_ added after emissions reached SS (6 and 21 afternoon); long clothing with O_3_ present from the start of the experiment (7 and 22); short clothing before O_3_ addition (8 and 23 morning); short clothing with O_3_ added after emissions reached SS (8 and 23 afternoon); and short clothing with O_3_ present from the start of the experiment (9 and 24).

With O_3_ present, the measured total OH reactivity for the same group of volunteers ranged between 25 ± 2 and 35 ± 0.2 s^–1^ (mean value across replicates ± 1 standard deviation). In the absence of O_3_, the OH reactivity was smaller (8 ± 3 to 11 ± 7 s^–1^ for short and long clothing) consistent with the experiments examined in Wang et al.^34^ Differences in OH reactivity for O_3_-free conditions are attributed primarily to variability in isoprene (C_5_H_8_) emission rates^33^ (differences in measured OH reactivity up to 24% and in calculated OH reactivity up to 22%). In the presence of O_3_, the measured OH reactivity between replicates agree within 1–18%. A similar agreement was observed for the respective calculated values (agreement within 1–18%, uncertainty on measurements ∼48%, uncertainty of calculations ∼30%). The replicates suggest that small differences in VOC emissions were present within the same group of volunteers on different experimental days.

For both clothing conditions, an increasing trend of OH reactivity was observed when O_3_ was introduced after steady-state conditions had been reached (29 ± 5 and 35 ± 0.2 s^–1^, mean measured OH reactivity for long and short clothing and standard deviation among replicates, respectively), compared to when O_3_ was introduced from the start of the experiment (25 ± 1.5 and 31 ± 2.4 s^–1^, mean measured OH reactivity for long and short clothing, respectively); see Figure 1. Differences were statistically significant (*p* < 0.05, paired sample *t*-test) when occupants wore short clothing. With O_3_ present, the OH reactivity was significantly larger with short clothing compared to the condition when long clothing was worn (*p* < 0.05, paired sample *t*-test).

As shown in Figure 1, differences between the measured total OH reactivity and the summed OH reactivity from the individually measured compounds are not significant, meaning that for the investigated conditions the reactive VOC budget, within the margin of uncertainty, is closed. Figure S1 examines the OH reactivity per chemical compound class, divided into hydrocarbons, alcohols, carboxylic acids, aromatic oxygenated compounds, carbonyl compounds, other oxygenated compounds, nitrogen-containing compounds, and sulfur-containing compounds (Table S2). In the absence of O_3_, when occupants wore long clothing, hydrocarbons (mainly represented by isoprene) explained 79% of the total OH reactivity, followed by carbonyls (15.7%, mainly formaldehyde, acetaldehyde, 1,4-butanedial, 6-MHO, 4-OPA, and geranyl acetone), other oxygenated compounds (1.45%), aromatics (1%), alcohols (1%), sulfur-containing compounds (0.8%), carboxylic acids (0.7%), and nitrogen-containing compounds (0.4%). Small differences were reported in the absence of O_3_, when occupants wore short clothing (hydrocarbons, carbonyls, and nitrogen-containing compounds explained 76%, 17%, and 1.2% of the OH reactivity, respectively). In the presence of O_3_, and with occupants wearing long clothing, their OH reactivity was explained by carbonyls (59%), hydrocarbons (34%), others (2.8%), aromatics (1.7%), carboxylic acids (1%), sulfur-containing compounds (0.5%), alcohols (0.4%), and nitrogen-containing compounds (0.3%). When occupants were wearing short clothing, carbonyls explained 65% of the total OH reactivity, followed by hydrocarbons (27.4%), others (2.9%), aromatics (2%), carboxylic acids (1.2%), sulfur-containing compounds (0.8%), nitrogen-containing compounds (0.3%), and alcohols (0.3%).

**Figure 2:**
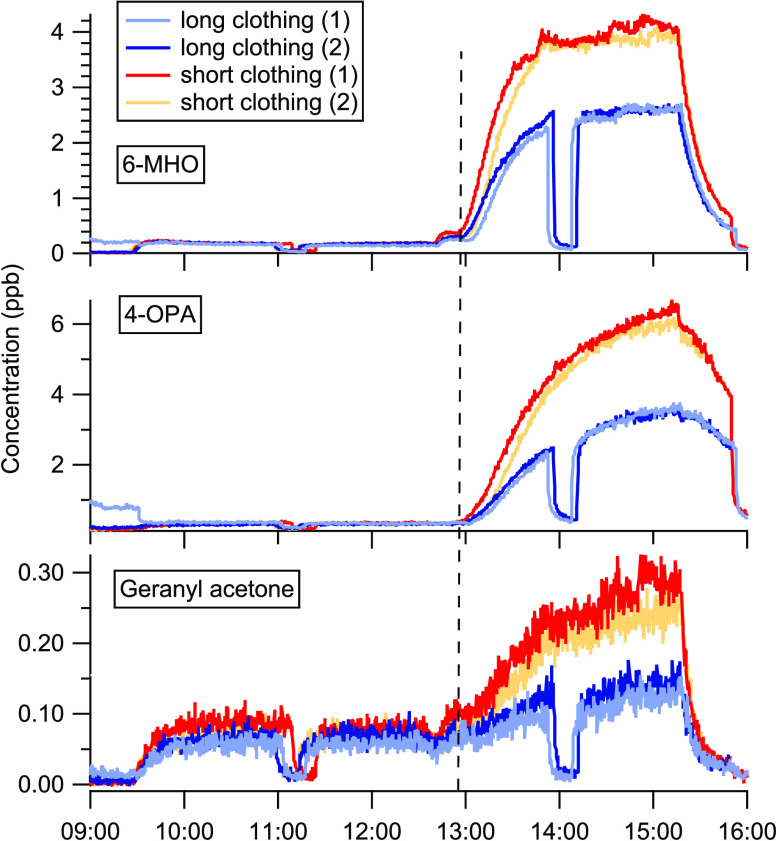
Concentrations of 6-MHO, 4-OPA, and geranyl acetone measured in the chamber occupied from 9:30 by four adults wearing long/short clothing. For each condition, two replicate experiments (*N* = 2) were conducted (long clothing conditions (6), (21), short clothing conditions (8), (23); see Table S1). The dashed line indicates when ozone was mixed into the chamber air. The dips correspond to measurements of the chamber supply air.

In terms of specific species, when long clothing was worn and O_3_ was present from the start of experiments, the compounds most influencing the reactivity were 6-MHO (C_8_H_14_O, 9.4 ± 2.2 s^–1^) and isoprene (C_5_H_8_, 9.3 ± 1.3 s^–1^), followed by 4-OPA (C_5_H_8_O_2_, 1.8 ± 0.4 s^–1^), 1,4-butanedial (C_4_H_6_O_2_, 1.4 ± 0.3 s^–1^), and acetaldehyde (C_2_H_4_O, 1.4 ± 0.2 s^–1^); values in parentheses correspond to the OH reactivity ± 1 standard deviation reported for the experimental replicates of the same condition (Table S3). In comparison, when O_3_ was added after the gas-phase concentration of occupant emitted compounds reached steady state (afternoon), compounds related to dermal emissions such as geranyl acetone (0.50 ± 0.06 s^–1^) and 6-MHO (9.91 ± 0.02 s^–1^) had a larger OH reactivity. Carbonyls had an even larger influence on the OH reactivity for occupants wearing short clothing: 6-MHO (14 ± 0.6 s^–1^) and isoprene (10.2 ± 0.8 s^–1^), followed by 4-OPA (3.2 ± 0.1 s^–1^), 1,4-butanedial (2 ± 0.03 s^–1^), and acetaldehyde (1.6 ± 0.03 s^–1^). In the case of occupants wearing short clothing when O_3_ was added in the afternoon, the OH reactivity of some carbonyl compounds, including 6-MHO (15.3 ± 0.4 s^–1^) and geranyl acetone (1 ± 0.1 s^–1^) further increased. Figure 2 shows the time series of 6-MHO, 4-OPA, and geranyl acetone concentrations. A 35–51% increase in the concentrations of those three compounds is associated with occupants wearing short rather than long clothing. In the presence of O_3_, clothing coverage of bare skin significantly influences the OH reactivity.

Depending on the clothing worn and the timing of the O_3_ introduction to the chamber, four cases can be distinguished: (i) clean long clothing worn just prior to O_3_ exposure (O_3_ from start), (ii) long clothing worn for ∼3 h prior to O_3_ exposure (O_3_ from SS), (iii) clean short clothing worn just prior to O_3_ exposure (O_3_ from start), and (iv) short clothing worn for ∼3 h prior to O_3_ exposure (O_3_ from SS). The O_3_ deposition velocity on the occupants (*ν*_occ_) was calculated for each of these cases to investigate whether the observed OH reactivity and VOC mixing ratios are compatible with bare skin-surface-to-clothed-surface area, and how clothing cleanliness influences emission (Table S4). Ozone deposition velocities calculated in our experiments are close to the upper limits of values previously reported in the literature^8,20^ for similar conditions. This is due to possibly higher air mixing achieved by the fans in the chamber. When O_3_ was added in the chamber from steady state in the afternoon (and occupants had been wearing the clothes for a longer time), O_3_ deposition velocity showed a small increase, while a larger increase and consistent trend was reported for k_occ_, VOC concentrations, and OH reactivity (Table S4). The yield was presumably enhanced by the presence of skin flakes or skin oil deposited on the clean clothing during the day, in agreement with the results reported by Lakey et al.^51^

In comparison, the O_3_ deposition velocity of four clean t-shirts (6.9 m/h) and four soiled t-shirts (9.4 m/h), exposed to O_3_ in the chamber are reported in Table S5. Results are in agreement with values reported in the literature^18,19,52^ but are substantially lower than those measured for the clothed occupants in this study. We observed very little difference in O_3_ removal for occupants wearing long or short clothing; therefore, the deposition velocity of clothing that is being worn is nearly the same as that of skin but higher than that of the clothing that was tested on its own. It is possible that the deposition velocity for clothing worn by people is higher because of enhanced convection around a body. The measured total OH reactivity of clean clothing was below the limit of detection (5 s^–1^) and of soiled clothing was 7 ± 3 s^–1^. The total OH reactivity of clothing and occupants depends on concentrations of the most reactive compounds generated from O_3_-initiated chemistry with skin oil. This chemistry depends on the O_3_ concentration inside the chamber and the mass-transport limited O_3_ flux to surfaces. Taking this into account, the OH reactivity is expected to scale with O_3_ concentration in an occupied, or recently occupied indoor environment.

### Effect of Temperature and Humidity

**Figure 3:**
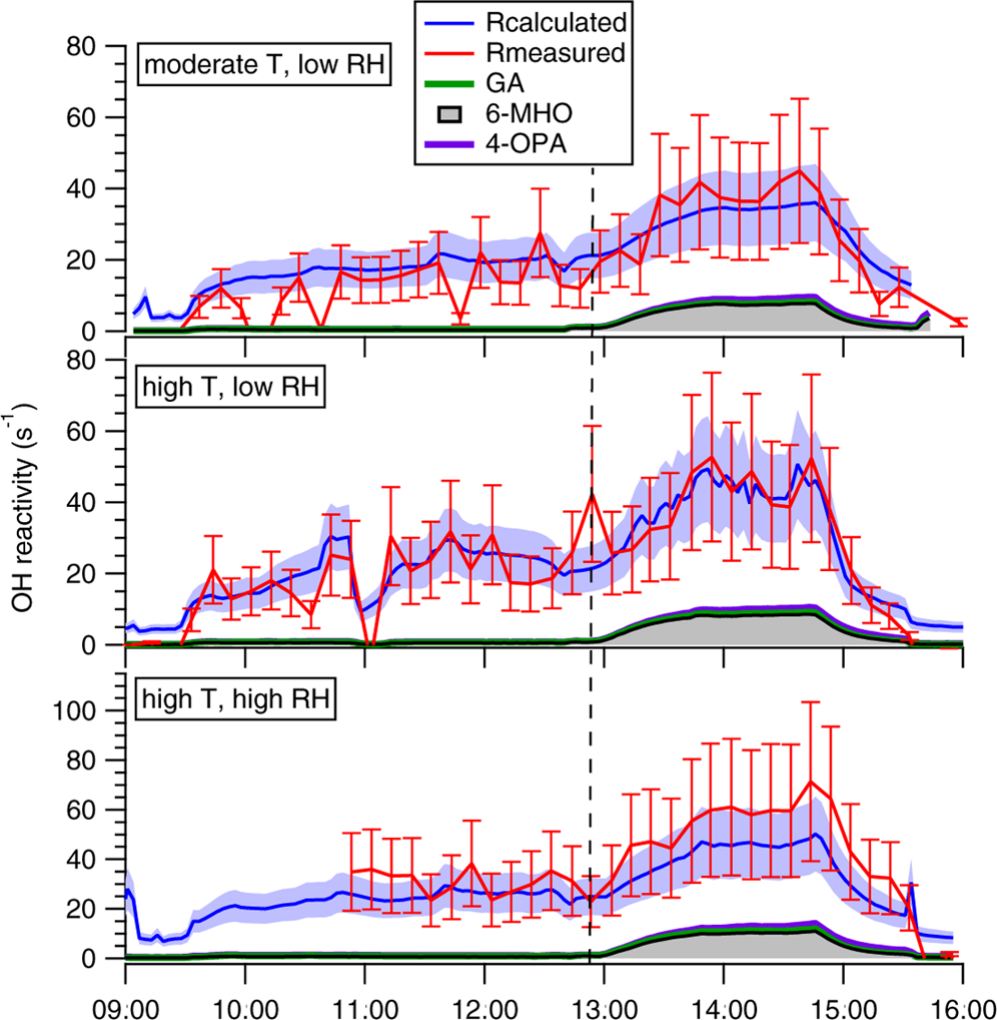
Measured and calculated OH reactivity from four adults occupying the chamber from 9:30, wearing long clothing, exposed to moderate or high temperature (*T*), and low or high humidity (RH). Ozone was added to the chamber air when occupant emissions reached steady state (dashed vertical line). Total measured and calculated OH reactivities are reported with their associated method uncertainties, 48 and 30%, respectively. 6-MHO, 4-OPA, and geranyl acetone (GA) OH reactivities are reported with the gray area and colored lines, respectively. Missing data points for the first 1.5 h in the bottom panel are due to instrument failure. The dip in the experiment at a high *T* and a low RH corresponds to the measurement of the chamber supply air.

To assess the effect of temperature and humidity, conditions (1), (4), and (2)^33^ (Table S1) are examined in Figure 3, for the measured total OH reactivity, summed calculated OH reactivity, 6-MHO, 4-OPA, and geranyl acetone OH reactivities. Specifically, four adult volunteers (group A1) occupied the chamber, wearing long clothing, at moderate air temperature (set point 25 °C) and low RH (set point 25%), at high temperature (set point 31 °C) and low RH, and at high temperature and RH (set point 65%); the actual temperatures were higher than the set points (see Table S1). In all cases, O_3_ was added to the chamber air after the occupant VOC emissions reached steady-state condition. Similar to the case of clothing, OH reactivity increased when O_3_ was present due to the presence of reactive carbonyls generated from skin ozonolysis reactions. With O_3_ present, the total OH reactivity measured at steady state (15 min before end of the exposure) varied between 41 s^–1^ (moderate temperature, low RH) and 63 s^–1^ (high temperature, high RH). For each positive change in condition (increased air temperature, increased relative humidity), higher concentrations of reactive compounds were present. In all three cases, the reactive VOC budget was closed within the margin of uncertainty (Figure 3).

**Figure 4:**
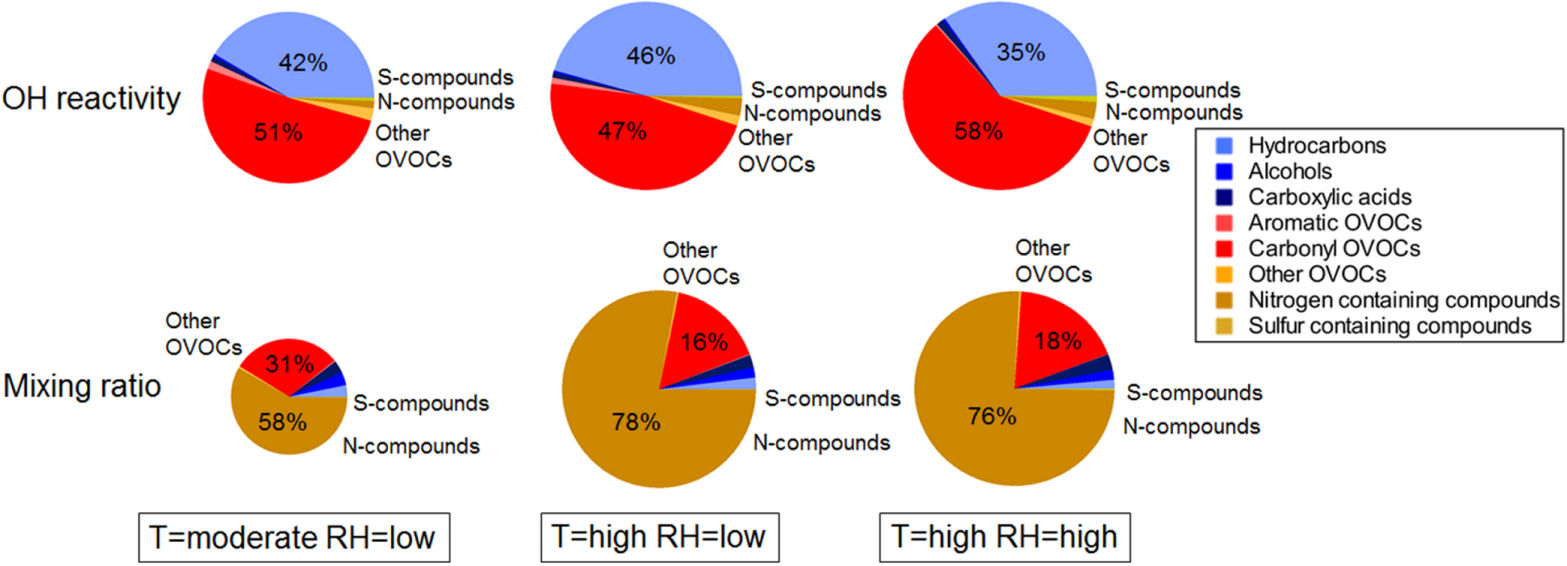
Speciated total OH reactivity (top row) and speciated total mixing ratios (bottom row) measured at the steady state with O_3_ present; four adults wearing long clothing occupied the climate chamber at various temperatures (*T*) and relative humidities (RH). Total OH reactivities represented in the pie charts in the top row are 32 ± 10, 41 ± 12, and 40 ± 12 s^–1^, for the three cases, moderate *T* and low RH, high *T* and low RH, and high *T* and high RH, respectively. Total mixing ratios represented in the pie charts in the bottom row are 190 ± 13, 405 ± 33, and 393 ± 32 ppb, for the three cases, moderate *T* and low RH, high *T* and low RH, and high *T* and high RH, respectively. The size of the pie charts is scaled to the total values, different scales are used between pies representing OH reactivity and pies representing the mixing ratios. Steady-state values were determined during the last 15 min before occupants left the chamber.

The classes of chemical compounds contributing to the total OH reactivity and their mixing ratios are shown in Figure 4. At a moderate temperature and a low RH, the dominant reactive compounds were carbonyls (51%), followed by hydrocarbons (41%), others (2.3%), aromatics (1.5%), nitrogen-containing compounds (1.4%, 96% of this fraction is represented by NH_3_), carboxylic acids (1%), sulfur compounds (0.6%), and alcohols (0.5%). In contrast to the OH reactivity, the total mixing ratio of the chemical compounds, which depends only on the abundance of the measured chemicals, is dominated by nitrogen-containing compounds, specifically by ammonia (NH_3_, 58%), carbonyl compounds (30%), alcohols (3.8%), carboxylic acids (3.4%), hydrocarbons (3.2%), others (0.5%), aromatics (0.3%), and sulfur compounds (0.2%). The reader should note that NH_3_ measurements were not available during the experiments involving group A2 (O_3_ and clothing effect). Results reported in Figure S1 (group A1) and Figure 4 (group A2) are in agreement, within the variability between groups observed in Wang et al.^34^

A small increase in temperature (at low RH), leaves the speciation of OH reactivity almost unchanged relative to the base case (the total hydrocarbon and carbonyl fractions OH reactivities increase, respectively, by 5.5 and 3 s^–1^). In contrast, the fractional mixing ratio is remarkably different, with the fraction of nitrogen-containing compounds (primarily NH_3_) increasing to 78%. The temperature-dependent emissions of ammonia were reported by Li et al.^43^ for the same experiments. The fractional contribution of other groups of compounds to the total mixing ratio decreased with increasing temperature (Figure 4). However, an increase in concentration was seen for geranyl acetone, a primary product of squalene ozonolysis, and 6-MHO, which is both a primary and secondary product of the reaction between squalene and O_3_ (Figure S2). Given the relatively small changes in absolute temperature in the chamber, changes in the gas-phase rate coefficients are anticipated to be small. Higher temperatures will increase the volatility of compounds from all surfaces in the chamber. However, human skin surface temperature changes are modulated metabolically. Increased sweat production will increase water vapor at the skin surface, favoring production of carbonyls over less volatile secondary ozonides.^24^ Further, sweat may alter the metabolic activity of the skin microbiome, resulting in a different suite of microbial emissions. At a high temperature and a high RH, carbonyl compounds dominated the total OH reactivity (58%), followed by hydrocarbons (35%), nitrogen-containing compounds (3%, of which 98% is NH_3_), others (1.3%), carboxylic acids (1.2%), S-compounds (1%), alcohols (0.4%), and aromatics (0.3%). The total concentration instead, was dominated by NH_3_ (75%, 99.5% of the total concentration of nitrogen-containing compounds), carbonyls (+3% compared to low RH), and carboxylic acids (+0.6%). With respect to the base case (moderate temperature, low RH), the steady-state 6-MHO concentration increased by 9% with the increase in temperature and by 27% with the increase in temperature and RH. Geranyl acetone concentration increased by 27% with the increase in temperature and 54% with the increase in temperature and RH. The 4-OPA concentration did not increase significantly (<1%) when temperature increased, while it increased by 34% when temperature and RH increased (Figure S2). The results from this study are in agreement with the study from Arata et al.^24^ for heterogeneous ozonolysis of squalene in a flow tube and skin oils on soiled t-shirts in the same climate chamber, where higher RH caused a greater yield of carbonyls from the Criegee intermediates of the squalene-O_3_ reaction.

### OH Reactivity-Influencing Factors

A dominance analysis was conducted to investigate the relative importance of the factors discussed in this study (indoor O_3_ concentration, clothing, temperature, and humidity) and in Wang et al. (age and group variability),^34^ for the total OH reactivity and individual OH reactivities of geranyl acetone, 6-MHO, and 4-OPA. The analysis aims at identifying the main drivers of human OH reactivity indoors through the relative importance of the target variables (see the Methods section). The OH reactivity data set comprises experiments conducted on whole body emissions of four volunteers occupying the chamber.^33^ The target factors varied in the following experimental ranges: indoor O_3_ concentration: 0–44.2 ppb; air temperature: 26–32.8 °C, air humidity: 18–63%, clothing coverage: short/long; age group: teenager/adult/senior; and five different groups of volunteers: A1, A2, A3, T4, S5.^33^

The dominance analysis of total OH reactivity (Figure S3) shows that O_3_ has the largest impact on the total OH reactivity (85%), followed by clothing (6%), variability between the groups of subjects occupying the chamber (group factor), temperature and humidity having comparable impact (2.5–3%), while the age of volunteers has a negligible impact in comparison. Ozone initiates surface reactions with skin lipids generating reactive VOCs whose OH reactivity is represented under the total OH reactivity term. When the same analysis is conducted without O_3_ as a target variable, the ranking of the other factors is preserved. The skin-related compounds, namely, geranyl acetone, 6-MHO, and 4-OPA, have OH reactivities influenced mostly by the factors O_3_ and clothing. 6-MHO, which is both a primary and secondary product of squalene ozonolysis, and 4-OPA, a secondary product only, are considerably more influenced by O_3_ than geranyl acetone, a primary product of squalene ozonolysis. Among clothing, temperature, and humidity, the analysis finds that clothing is the most important factor. In our experiments, the clothing effect was examined with group A2, while temperature and humidity were examined with group A1. Total OH reactivity of group A1 emissions was higher than the total OH reactivity of group A2 emissions, masking the relative importance of the clothing factor compared to humidity and temperature. The squalene reduction observed from the skin wipes sampled before and after each experiment from the individuals in groups A1 and A2 is in general agreement with the total OH reactivity. That is, a larger reduction in squalene is observed for the higher reactivity (up to 77% reduction of squalene for up to 63 s^–1^ OH reactivity), and a larger reduction is observed in A1 (68% on average) than A2 (20% on average).

These results highlight the importance of skin emissions and their ozonolysis products for the OH reactivity of human occupants indoors, their variability with temperature, relative humidity, and clothing, and ultimately their importance for indoor air quality.

Outdoor to indoor transport of O_3_ and exposed skin surface area are especially important factors. On a hot summer day spent indoors in a city with high concentrations of ground-level O_3_, more ventilation (increasing indoor O_3_), or less clothing (increasing O_3_ flux to bare skin) may be necessary to improve comfort. If more people occupy the same indoor environment, the effect on indoor air chemistry will be further amplified. 4-OPA and 6-MHO are known respiratory irritants,^53,54^ while the concomitant effect of exposure to multiple chemicals and the risk to certain population sectors is yet unknown.^55^ Remarkably, the indoor OH reactivity measured here for the first time was comparable to the OH reactivity measured in a variety of outdoor environments, including cities^56−58^ and forests.^58,59^

### Current Results in Relation to the Findings from Wang et al. and Previous Studies

Results reported in Wang et al.^34^ comprised experiments (1), (6), (21), (10), (18), (26), (16), (25), (12), and (13); results from the current study comprise experiments (1), (2), (4), (6), (7), (8), (9), (21), (22), (23), and (24).^33^ Recall that Wang et al.^34^ analyzed the OH reactivity of isolated breath and dermal emissions as well as whole-body emissions from volunteers of different age groups in the presence and absence of ozone and found that the OH reactivity could be accounted for by the trace gases measured and that it was sensitive to ozone but not to age. The current study focuses on the impact of varied environmental factors on two groups of adult volunteers, as well as reproducibility for sets of duplicated experiments. Results from these experiments have not been previously reported and are particularly relevant to the dependency of human OH reactivity on the absence or presence of O_3_, and the change in the OH reactivity of human emissions with changes in skin coverage by clothing, air temperature, and relative humidity. Skin coverage by clothing and worn/soiled clothing play an important role in the ozonolysis of skin-oil constituents. The role of clothing has been previously investigated in a number of O_3_ and VOCs experiments,^18,21,52,60^ modeling studies^19,51^ and now through measurements of occupant OH reactivity (this work). Additionally, to our knowledge, this is the first study reporting the effect of temperature on occupant VOC emissions and their OH reactivity. The experimental reproducibility of OH reactivity measurements across replicated experiments (6–21, 7–22, 8–23, and 9–24) reported in this work demonstrates that this technique can be used with confidence in indoor air studies. With O_3_ present, the standard deviation among steady-state values was <18%, and the method uncertainty was ∼48%. With O_3_ absent, the mean measured OH reactivity was 10 ± 2 s^–1^ and the standard deviation among the steady-state reactivity values was 24%, which is mainly explained by the increased uncertainty of the method for measuring OH reactivity when values are close to the instrumental limit of detection.^42^

## Supporting Information Available

Experimental conditions included in this study (Table S1); volatile organic compounds and their classification in compounds classes used to determine the summed calculated OH reactivity (Table S2); speciated OH reactivity from occupants wearing long clothing and short clothing, before ozone exposure, with ozone added at the steady state of emissions (afternoon), and with ozone added from the beginning of the experiment (Figure S1); 10 most reactive volatile organic compounds emitted by four adult volunteers wearing long clothing and short clothing exposed to ozone from start of the experiment and in the afternoon, from SS of the emissions (Table S3); calculated O_3_ deposition velocity on four occupants (*v*_occ_), first-order rate constant for O_3_ loss (*k*_d_), measured 6-MHO, 4-OPA, geranyl acetone concentrations, and measured total OH reactivity at SS for each experimental condition (Table S4); ozone deposition velocity and OH reactivity of four clean and soiled (worn overnight ∼8 h) t-shirts, and corresponding parameters used (Table S5); 10 most reactive volatile organic compounds emitted by four adult volunteers wearing long clothing exposed to ozone, at moderate temperature and low RH, high temperature and low RH, and high temperature and high RH (Table S6); concentrations of 6-MHO, 4-OPA, and geranyl acetone measured in the chamber occupied by four adults from 9:30, wearing long clothing, and exposed to low/high temperature and low/high relative humidity (Figure S2); dominance analysis for the total OH reactivity of human beings, with varying conditions of ozone exposure, short/long clothing, volunteers groups, relative humidity, temperature, and age of volunteers (teens/adults/seniors) (Figure S3) (PDF)

## References

[ref1] Klepeis N. E.; Nelson W. C.; Ott W. R.; Robinson J. P.; Tsang A. M.; Switzer P.; Behar J. V.; Hern S. C.; Engelmann W. H. The National Human Activity Pattern Survey (NHAPS): A Resource for Assessing Exposure to Environmental Pollutants. J. Exposure Sci. Environ. Epidemiol. 2001, 11, 231–252. 10.1038/sj.jea.7500165. 11477521

[ref2] Press Corner: https://ec.europa.eu/commission/presscorner/home/en (accessed Jan 24, 2021).

[ref3] US EPA, O. Introduction to Indoor Air Quality https://www.epa.gov/indoor-air-quality-iaq/introduction-indoor-air-quality (accessed Jan 24, 2021).

[ref4] Ault A. P.; Grassian V. H.; Carslaw N.; Collins D. B.; Destaillats H.; Donaldson D. J.; Farmer D. K.; Jimenez J. L.; McNeill V. F.; Morrison G. C.; O’Brien R. E.; Shiraiwa M.; Vance M. E.; Wells J. R.; Xiong W. Indoor Surface Chemistry: Developing a Molecular Picture of Reactions on Indoor Interfaces. Chem 2020, 6, 3203–3218. 10.1016/j.chempr.2020.08.023. 32984643 PMC7501779

[ref5] Tang X.; Misztal P. K.; Nazaroff W. W.; Goldstein A. H. Volatile Organic Compound Emissions from Humans Indoors. Environ. Sci. Technol. 2016, 50, 12686–12694. 10.1021/acs.est.6b04415. 27934268

[ref6] Weschler C. J. Roles of the Human Occupant in Indoor Chemistry. Indoor Air 2016, 26, 6–24. 10.1111/ina.12185. 25607256

[ref7] de Lacy Costello B.; Amann A.; Al-Kateb H.; Flynn C.; Filipiak W.; Khalid T.; Osborne D.; Ratcliffe N. M. A Review of the Volatiles from the Healthy Human Body. J. Breath Res. 2014, 8, 014001 10.1088/1752-7155/8/1/014001. 24421258

[ref8] Wisthaler A.; Weschler C. J. Reactions of Ozone with Human Skin Lipids: Sources of Carbonyls, Dicarbonyls, and Hydroxycarbonyls in Indoor Air. Proc. Natl. Acad. Sci. USA 2010, 107, 6568–6575. 10.1073/pnas.0904498106. 19706436 PMC2872416

[ref9] Lakey P. S. J.; Wisthaler A.; Berkemeier T.; Mikoviny T.; Pöschl U.; Shiraiwa M. Chemical Kinetics of Multiphase Reactions between Ozone and Human Skin Lipids: Implications for Indoor Air Quality and Health Effects. Indoor Air 2017, 27, 816–828. 10.1111/ina.12360. 27943451

[ref10] Kruza M.; Carslaw N. How Do Breath and Skin Emissions Impact Indoor Air Chemistry?. Indoor Air 2019, 29, 369–379. 10.1111/ina.12539. 30663813

[ref11] Amann A.; Costello B.; Miekisch W.; Schubert J.; Buszewski B.; Pleil J.; Ratcliffe N.; Risby T. The Human Volatilome: Volatile Organic Compounds (VOCs) in Exhaled Breath, Skin Emanations, Urine, Feces and Saliva. J. Breath Res. 2014, 8, 034001 10.1088/1752-7155/8/3/034001. 24946087

[ref12] Furukawa S.; Sekine Y.; Kimura K.; Umezawa K.; Asai S.; Miyachi H. Simultaneous and Multi-Point Measurement of Ammonia Emanating from Human Skin Surface for the Estimation of Whole Body Dermal Emission Rate. J. Chromatogr. B 2017, 1053, 60–64. 10.1016/j.jchromb.2017.03.034. 28411465

[ref13] Weschler C. J.; Carslaw N. Indoor Chemistry. Environ. Sci. Technol. 2018, 52, 2419–2428. 10.1021/acs.est.7b06387. 29402076

[ref14] Grøntoft T.; Raychaudhuri M. R. Compilation of Tables of Surface Deposition Velocities for O3, NO2 and SO2 to a Range of Indoor Surfaces. Atmos. Environ. 2004, 38, 533–544. 10.1016/j.atmosenv.2003.10.010.

[ref15] Morrison G. C.; Nazaroff W. W. The Rate of Ozone Uptake on Carpets: Experimental Studies. Environ. Sci. Technol. 2000, 34, 4963–4968. 10.1021/es001361h.

[ref16] Gall E.; Darling E.; Siegel J. A.; Morrison G. C.; Corsi R. L. Evaluation of Three Common Green Building Materials for Ozone Removal, and Primary and Secondary Emissions of Aldehydes. Atmos. Environ. 2013, 77, 910–918. 10.1016/j.atmosenv.2013.06.014.

[ref17] Bako-Biro Z.; Weschler C. J.; Wargocki P.; Fanger P. O. Effects of Indoor Pollution Sources and Ventilation Rate on Ozone Surface Removal Rate and the Occurrence of Oxygenated VOCs in an Office Space. In 10th International Conference on Indoor Air Quality and Climate, 2005; pp 2320–2324.

[ref18] Tamás G.; Weschler C. J.; Bakó-Biró Z.; Wyon D. P.; Strøm-Tejsen P. Factors Affecting Ozone Removal Rates in a Simulated Aircraft Cabin Environment. Atmos. Environ. 2006, 40, 6122–6133. 10.1016/j.atmosenv.2006.05.034.

[ref19] Rim D.; Gall E. T.; Ananth S.; Won Y. Ozone Reaction with Human Surfaces: Influences of Surface Reaction Probability and Indoor Air Flow Condition. Build. Environ. 2018, 130, 40–48. 10.1016/j.buildenv.2017.12.012.

[ref20] Pandrangi L. S.; Morrison G. C. Ozone Interactions with Human Hair: Ozone Uptake Rates and Product Formation. Atmos. Environ. 2008, 42, 5079–5089. 10.1016/j.atmosenv.2008.02.009.

[ref21] Coleman B. K.; Destaillats H.; Hodgson A. T.; Nazaroff W. W. Ozone Consumption and Volatile Byproduct Formation from Surface Reactions with Aircraft Cabin Materials and Clothing Fabrics. Atmos. Environ. 2008, 42, 642–654. 10.1016/j.atmosenv.2007.10.001.

[ref22] Nagda N.; Nazaroff W.; Gadgil A.; Weschler C. STP13101S Modeling of Indoor Air Quality and Exposure; ASTM International: West Conshohocken, PA, 1993.

[ref23] Yang S.; Gao K.; Yang X. Volatile Organic Compounds (VOCs) Formation Due to Interactions between Ozone and Skin-Oiled Clothing: Measurements by Extraction-Analysis-Reaction Method. Build. Environ. 2016, 103, 146–154. 10.1016/j.buildenv.2016.04.012.

[ref24] Arata C.; Heine N.; Wang N.; Misztal P. K.; Wargocki P.; Bekö G.; Williams J.; Nazaroff W. W.; Wilson K. R.; Goldstein A. H. Heterogeneous Ozonolysis of Squalene: Gas-Phase Products Depend on Water Vapor Concentration. Environ. Sci. Technol. 2019, 53, 14441–14448. 10.1021/acs.est.9b05957. 31757120

[ref25] Abbatt J. P. D.; Wang C. The Atmospheric Chemistry of Indoor Environments. Environ. Sci.: Processes Impacts 2020, 22, 25–48. 10.1039/C9EM00386J. 31712796

[ref26] Williams J.; Brune W. A Roadmap for OH Reactivity Research. Atmos. Environ. 2015, 106, 371–372. 10.1016/j.atmosenv.2015.02.017.

[ref27] Nölscher A. C.; Yañez-Serrano A. M.; Wolff S.; de Araujo A. C.; Lavrič J. V.; Kesselmeier J.; Williams J. Unexpected Seasonality in Quantity and Composition of Amazon Rainforest Air Reactivity. Nat. Commun. 2016, 7, 10383 10.1038/ncomms10383. 26797390 PMC4735797

[ref28] Fuchs H.; Tan Z.; Lu K.; Bohn B.; Broch S.; Brown S. S.; Dong H.; Gomm S.; Häseler R.; He L.; Hofzumahaus A.; Holland F.; Li X.; Liu Y.; Lu S.; Min K.-E.; Rohrer F.; Shao M.; Wang B.; Wang M.; Wu Y.; Zeng L.; Zhang Y.; Wahner A.; Zhang Y. OH Reactivity at a Rural Site (Wangdu) in the North China Plain: Contributions from OH Reactants and Experimental OH Budget. Atmos. Chem. Phys. 2017, 17, 645–661. 10.5194/acp-17-645-2017.

[ref29] Zannoni N.; Gros V.; Sarda Esteve R.; Kalogridis C.; Michoud V.; Dusanter S.; Sauvage S.; Locoge N.; Colomb A.; Bonsang B. Summertime OH Reactivity from a Receptor Coastal Site in the Mediterranean Basin. Atmos. Chem. Phys. 2017, 17, 12645–12658. 10.5194/acp-17-12645-2017.

[ref30] Kumar V.; Chandra B. P.; Sinha V. Large Unexplained Suite of Chemically Reactive Compounds Present in Ambient Air Due to Biomass Fires. Sci. Rep. 2018, 8, 626 10.1038/s41598-017-19139-3. 29330495 PMC5766614

[ref31] Whalley L.; Stone D.; Heard D. New Insights into the Tropospheric Oxidation of Isoprene: Combining Field Measurements, Laboratory Studies, Chemical Modelling and Quantum Theory. Top. Curr. Chem. 2014, 339, 55–95. 10.1007/128_2012_359. 22955507

[ref32] Pfannerstill E. Y.; Wang N.; Edtbauer A.; Bourtsoukidis E.; Crowley J. N.; Dienhart D.; Eger P. G.; Ernle L.; Fischer H.; Hottmann B.; Paris J.-D.; Stönner C.; Tadic I.; Walter D.; Lelieveld J.; Williams J. Shipborne Measurements of Total OH Reactivity around the Arabian Peninsula and Its Role in Ozone Chemistry. Atmos. Chem. Phys. 2019, 19, 11501–11523. 10.5194/acp-19-11501-2019.

[ref33] Bekö G.; Wargocki P.; Wang N.; Li M.; Weschler C. J.; Morrison G.; Langer S.; Ernle L.; Licina D.; Yang S.; Zannoni N.; Williams J. The Indoor Chemical Human Emissions and Reactivity (ICHEAR) Project: Overview of Experimental Methodology and Preliminary Results. Indoor Air 2020, 30, 1213–1228. 10.1111/ina.12687. 32424858

[ref34] Wang N.; Zannoni N.; Ernle L.; Bekö G.; Wargocki P.; Li M.; Weschler C. J.; Williams J. Total OH Reactivity of Emissions from Humans: In Situ Measurement and Budget Analysis. Environ. Sci. Technol. 2021, 55, 149–159. 10.1021/acs.est.0c04206. 33295177 PMC7788569

[ref35] Salvador C. M.; Bekö G.; Weschler C. J.; Morrison G.; Le Breton M.; Hallquist M.; Ekberg L.; Langer S. Indoor Ozone/Human Chemistry and Ventilation Strategies. Indoor Air 2019, 29, 913–925. 10.1111/ina.12594. 31420890 PMC6856811

[ref36] Fuchs H.; Novelli A.; Rolletter M.; Hofzumahaus A.; Pfannerstill E. Y.; Kessel S.; Edtbauer A.; Williams J.; Michoud V.; Dusanter S.; Locoge N.; Zannoni N.; Gros V.; Truong F.; Sarda-Esteve R.; Cryer D. R.; Brumby C. A.; Whalley L. K.; Stone D.; Seakins P. W.; Heard D. E.; Schoemaecker C.; Blocquet M.; Coudert S.; Batut S.; Fittschen C.; Thames A. B.; Brune W. H.; Ernest C.; Harder H.; Muller J. B. A.; Elste T.; Kubistin D.; Andres S.; Bohn B.; Hohaus T.; Holland F.; Li X.; Rohrer F.; Kiendler-Scharr A.; Tillmann R.; Wegener R.; Yu Z.; Zou Q.; Wahner A. Comparison of OH Reactivity Measurements in the Atmospheric Simulation Chamber SAPHIR. Atmos. Meas. Tech. 2017, 10, 4023–4053. 10.5194/amt-10-4023-2017.

[ref37] Sinha V.; Williams J.; Crowley J. N.; Lelieveld J. The Comparative Reactivity Method – a New Tool to Measure Total OH Reactivity in Ambient Air. Atmos. Chem. Phys. 2008, 8, 2213–2227. 10.5194/acp-8-2213-2008.

[ref38] Lindinger W.; Jordan A. Proton-Transfer-Reaction Mass Spectrometry (PTR–MS): On-Line Monitoring of Volatile Organic Compounds at Pptv Levels. Chem. Soc. Rev. 1998, 27, 347–375. 10.1039/a827347z.

[ref39] Atkinson R.; Aschmann S. M.; Winer A. M.; Carter W. P. L. Rate Constants for the Gas Phase Reactions of OH Radicals and O3 with Pyrrole at 295 ± 1 K and Atmospheric Pressure. Atmos. Environ. (1967) 1984, 18, 2105–2107. 10.1016/0004-6981(84)90196-3.

[ref40] Dillon T. J.; Tucceri M. E.; Dulitz K.; Horowitz A.; Vereecken L.; Crowley J. N. Reaction of Hydroxyl Radicals with C4H5N (Pyrrole): Temperature and Pressure Dependent Rate Coefficients. J. Phys. Chem. A 2012, 116, 6051–6058. 10.1021/jp211241x. 22264135

[ref41] Zannoni N.; Dusanter S.; Gros V.; Sarda Esteve R.; Michoud V.; Sinha V.; Locoge N.; Bonsang B. Intercomparison of Two Comparative Reactivity Method Instruments Inf the Mediterranean Basin during Summer 2013. Atmos. Meas. Tech. 2015, 8, 3851–3865. 10.5194/amt-8-3851-2015.

[ref42] Michoud V.; Hansen R. F.; Locoge N.; Stevens P. S.; Dusanter S. Detailed Characterizations of the New Mines Douai Comparative Reactivity Method Instrument via Laboratory Experiments and Modeling. Atmos. Meas. Tech. 2015, 8, 3537–3553. 10.5194/amt-8-3537-2015.

[ref43] Li M.; Weschler C. J.; Bekö G.; Wargocki P.; Lucic G.; Williams J. Human Ammonia Emission Rates under Various Indoor Environmental Conditions. Environ. Sci. Technol. 2020, 54, 5419–5428. 10.1021/acs.est.0c00094. 32233434

[ref44] Zhao J.; Zhang R. Proton Transfer Reaction Rate Constants between Hydronium Ion (H3O+) and Volatile Organic Compounds. Atmos. Environ. 2004, 38, 2177–2185. 10.1016/j.atmosenv.2004.01.019.

[ref45] Bourtsoukidis E.; Helleis F.; Tomsche L.; Fischer H.; Hofmann R.; Lelieveld J.; Williams J. An Aircraft Gas Chromatograph–Mass Spectrometer System for Organic Fast Identification Analysis (SOFIA): Design, Performance and a Case Study of Asian Monsoon Pollution Outflow. Atmos. Meas. Tech. 2017, 10, 5089–5105. 10.5194/amt-10-5089-2017.

[ref46] Du Bois D.; Du Bois E. F. A Formula to Estimate the Approximate Surface Area If Height and Weight Be Known. Nutrition 1989, 5, 303–311. 2520314

[ref47] US EPA. National Center for Environmental Assessment, W. D. Exposure Factors Handbook 2011th ed. (Final Report). https://cfpub.epa.gov/ncea/risk/recordisplay.cfm?deid=236252 (accessed March 12, 2021).

[ref48] Azen R.; Budescu D. V. The Dominance Analysis Approach for Comparing Predictors in Multiple Regression. Psychol. Methods 2003, 8, 129–148. 10.1037/1082-989X.8.2.129. 12924811

[ref49] Azen R.; Budescu D. V. Comparing Predictors in Multivariate Regression Models: An Extension of Dominance Analysis. J. Educ. Behav. Stat. 2006, 31, 157–180. 10.3102/10769986031002157.

[ref50] Azen R.; Budescu D. V.; Reiser B. Criticality of Predictors in Multiple Regression. Br. J. Math. Stat. Psychol. 2001, 54, 201–225. 10.1348/000711001159483. 11817090

[ref51] Lakey P. S. J.; Morrison G. C.; Won Y.; Parry K. M.; von Domaros M.; Tobias D. J.; Rim D.; Shiraiwa M. The Impact of Clothing on Ozone and Squalene Ozonolysis Products in Indoor Environments. Commun. Chem. 2019, 2, 56 10.1038/s42004-019-0159-7.

[ref52] Rai A. C.; Guo B.; Lin C.-H.; Zhang J.; Pei J.; Chen Q. Ozone Reaction with Clothing and Its Initiated VOC Emissions in an Environmental Chamber. Indoor Air 2014, 24, 49–58. 10.1111/ina.12058. 23841649

[ref53] Anderson S. E.; Franko J.; Jackson L. G.; Wells J. R.; Ham J. E.; Meade B. J. Irritancy and Allergic Responses Induced by Exposure to the Indoor Air Chemical 4-Oxopentanal. Toxicol. Sci. 2012, 127, 371–381. 10.1093/toxsci/kfs102. 22403157 PMC3355310

[ref54] Wolkoff P. Indoor Air Pollutants in Office Environments: Assessment of Comfort, Health, and Performance. Int. J. Hyg. Environ. Health 2013, 216, 371–394. 10.1016/j.ijheh.2012.08.001. 22954455

[ref55] Mitchell C. S.; Zhang J. J.; Sigsgaard T.; Jantunen M.; Lioy P. J.; Samson R.; Karol M. H. Current State of the Science: Health Effects and Indoor Environmental Quality. Environ. Health Perspect. 2007, 115, 958–964. 10.1289/ehp.8987. 17589607 PMC1892137

[ref56] Dolgorouky C.; Gros V.; Sarda-Esteve R.; Sinha V.; Williams J.; Marchand N.; Sauvage S.; Poulain L.; Sciare J.; Bonsang B. Total OH Reactivity Measurements in Paris during the 2010 MEGAPOLI Winter Campaign. Atmos. Chem. Phys. 2012, 12, 9593–9612. 10.5194/acp-12-9593-2012.

[ref57] Ren X. HOx Concentrations and OH Reactivity Observations in New York City during PMTACS-NY2001. Atmos. Environ. 2003, 37, 3627–3637. 10.1016/S1352-2310(03)00460-6.

[ref58] Williams J.; Keßel S. U.; Nölscher A. C.; Yang Y.; Lee Y.; Yáñez-Serrano A. M.; Wolff S.; Kesselmeier J.; Klüpfel T.; Lelieveld J.; Shao M. Opposite OH Reactivity and Ozone Cycles in the Amazon Rainforest and Megacity Beijing: Subversion of Biospheric Oxidant Control by Anthropogenic Emissions. Atmos. Environ. 2016, 125, 112–118. 10.1016/j.atmosenv.2015.11.007.

[ref59] Zannoni N.; Gros V.; Lanza M.; Sarda R.; Bonsang B.; Kalogridis C.; Preunkert S.; Legrand M.; Jambert C.; Boissard C.; Lathiere J. OH Reactivity and Concentrations of Biogenic Volatile Organic Compounds in a Mediterranean Forest of Downy Oak Trees. Atmos. Chem. Phys. 2016, 16, 1619–1636. 10.5194/acp-16-1619-2016.

[ref60] Wisthaler A.; Tamás G.; Wyon D. P.; Strøm-Tejsen P.; Space D.; Beauchamp J.; Hansel A.; Märk T. D.; Weschler C. J. Products of Ozone-Initiated Chemistry in a Simulated Aircraft Environment. Environ. Sci. Technol. 2005, 39, 4823–4832. 10.1021/es047992j. 16053080

