# Supplementary material for: Effect of Ozone, Clothing, Temperature, and Humidity on the Total OH Reactivity Emitted from Humans
Source: Environ Sci Technol. 2021 Sep 30;55(20):13614–24. doi: 10.1021/acs.est.1c01831 (PMC8529706; doi:10.1021/acs.est.1c01831)
Supplement: Supplementary file 1 — es1c01831_si_001.pdf [file es1c01831_si_001.pdf]

Supplementary material for:

The effect of ozone, clothing, temperature and humidity on the total OH reactivity emitted from humans

Nora Zannoni[1], Mengze Li[1], Nijing Wang[1], Lisa Ernle[1], Gabriel Bekö[2], Pawel Wargocki[2], Sarka Langer[3,4], Charles J. Weschler[2,5], Glenn Morrison[6], Jonathan Williams[1].*

[1]Atmospheric Chemistry Department, Max Planck Institute for Chemistry, Mainz, Germany

[2]International Centre for Indoor Environment and Energy, Department of Civil Engineering, Technical University of Denmark, Lyngby, Denmark

[3]IVL Swedish Environmental Research Institute, Göteborg, Sweden

[4]Division of Building Services Engineering, Department of Architecture and Civil Engineering, Chalmers University of Technology, Göteborg, Sweden

[5]Environmental and Occupational Health Sciences Institute, Rutgers University, New Jersey, United States

[6]Department of Environmental Sciences and Engineering, Gillings School of Global Public Health, The University of North Carolina at Chapel Hill, North Carolina, United States

*corresponding author

Table S1. Experimental conditions included in this study. All experiments were conducted on four adult volunteers. Experiments 21-24 are replicates of 6-9. Detailed information is available in Bekö et al.,[1].*

Experiment	clothing	Mean air temperature at steady state O_3 [°C]	Mean air humidity at steady state O_3 [%]	Ozone present (SS-steady state)	Inlet Ozone concentration [ppb]	Chamber Ozone concentration [ppb]
1	long	30.3	32	From SS	98.4	34.1
2	long	32.3	63	From SS	125.6	38.8
4	long	31.8	30	From SS	98.3	33.6
6	long	27.5	18	From SS	105.9	36.3
21	long	27.7	21	From SS	100.3	35.4
7	long	26	27	From start	96.8	34.8
22	long	27.1	20	From start	104.5	39.6
8	short	28.4	23	From SS	102.5	35
23	short	28.9	22	From SS	102.8	34.5
9	short	26.5	20	From start	100.8	36.9
24	short	27.1	19	From start	102.2	36.3

* Experiment numbers are identical to those in Bekö et al.,[1]. Experiments 1, 2, and 4 were with volunteer Group A1. Experiments 6, 21, 7, 22, 8, 23, 9, and 24 were with volunteer Group A2.

Table S2. Compound assigned identities and their classification in compounds classes used to determine the summed calculated OH reactivity[2]. *m/z* refers to the protonated mass measured by PTR-Tof-MS. Rate coefficients k_{i+OH} of *i*+OH were considered at 298 K.

Compound group	*m/z*	Formula	ID compound	k_{i+OH} (cm^3 molecules^{-1} s^{-1})	Refs.
Hydrocarbons (HC)		C5H8	isoprene[a]	1.00×10^{-10}	3
	79.053	C6H6	benzene[b]	1.20×10^{-12}	3
	93.070	C7H8	toluene[b]	5.60×10^{-12}	3
	105.070	C8H8	styrene	5.80×10^{-11}	4
	107.086	C8H10	xylene[b]	1.70×10^{-11}	4
	119.086	C9H10	2-phenylpropene	5.30×10^{-11}	5
	121.101	C9H12	trimethylbenzene[b]	4.06×10^{-11}	4
	133.101	C10H12	benzene,(2-methyl-1-propenyl)-	3.30×10^{-11}	5
	137.132	C10H16	limonene[b]	1.64×10^{-10}	3
OVOC Alcohols	33.034	CH4O	methanol[b]	9.00×10^{-13}	3
	47.049	C2H6O	ethanol	3.20×10^{-12}	3
OVOC Acids	43.018<br>61.029	C2H4O2	acetic acid	8.00×10^{-13}	6
	73.029	C3H4O2	acrylic acid	1.75×10^{-11}	7
	89.023	C3H4O3	pyruvic acid	1.24×10^{-13}	8
OVOC Aromatics	95.049	C6H6O	phenol	3.27×10^{-11}	9
	107.049	C7H6O	benzaldehyde	1.20×10^{-11}	3
	109.029	C6H4O2	1,4-benzoquinone	4.60×10^{-12}	10
	109.065	C7H8O	methylphenol/methoxybenzene/toluene-1,2-oxide 3/2-methyloxepin[c]	7.74×10^{-11}	11-13

OVOC Aromatics	121.065	C8H8O	tolualdehyde	1.60×10^{-11}	4
	123.044	C7H6O2	p-benzoquinone, 2-methyl-	2.35×10^{-11}	10
	123.081	C8H10O	dimethylphenol	8.48×10^{-11}	12
	125.060	C7H8O2	4-methoxyphenol /3-methoxyphenol/2-methoxyphenol[c]	8.94×10^{-11}	12
	135.081	C9H10O	dimethylbenzaldehyde[c]	2.74×10^{-11}	14
	137.060	C8H8O2	1,4-benzodioxane	2.52×10^{-11}	15
	149.096	C10H12O	2,4,5-trimethylbenzaldehyde	4.27×10^{-11}	16
OVOC Carbonyls	31.018	CH2O	formaldehyde	9.37×10^{-12}	4
	45.033	C2H4O	acetaldehyde	1.50×10^{-11}	3
	57.034	C3H4O	acrolein/2-propenal[c]	2.00×10^{-11}	17
	59.048	C3H6O	acetone[b]	1.80×10^{-13}	3
	71.048	C4H6O	methyl vinyl ketone (MVK)/methacrolein (MACR)/isoprene hydroxy hydroperoxide (ISOPOOH) [b]	2.45×10^{-11}	3
	73.064	C4H8O	methyl ethyl ketone[b]	1.10×10^{-12}	3
	75.044	C3H6O2	hydroxyacetone	3.00×10^{-12}	18
	83.049	C5H6O	4-oxopentanal fragment; methylfuran[d]	2.00×10^{-11}	19
	85.028	C4H4O2	butenedial	5.21×10^{-11}	20
	85.065	C5H8O	2-pentenal	4.37×10^{-11}	21
	87.044	C4H6O2	1,4-butanedial[e]	5.70×10^{-11}	22
	87.081	C5H10O	pentanal	2.80×10^{-11}	4
	97.020	C5H4O2	2 or 3-furancarboxaldehyde[c]	4.18×10^{-11}	23
	97.065	C6H8O	2,4-hexadienal	5.90×10^{-11}	24
	99.045	C5H6O2	4-oxo-2-pentenal	5.58×10^{-11}	20
	99.081	C6H10O	cis-3-hexenal; (2e)-2-hexenal; 2-pentenal, 2-methyl-[c]	4.64×10^{-11}	25-27
	101.060	C5H8O2	4-oxopentanal (4-OPA)	2.00×10^{-11}	19
	101.096	C6H12O	hexanal	3.00×10^{-11}	4
	103.075	C5H10O2	1-hydroxy-2-methyl-3-butanone	1.62×10^{-11}	28
	109.101 127.112	C8H14O	6-methyl-5-hepten-2-one (6-MHO)	1.57×10^{-10}	29
	111.081	C7H10O	4-methylenehex-5-enal/(3z)-4-methylhexa-3,5-dienal/(3e)-4-methylhexa-3,5-dienal/4-methylcyclohex-3-en-1-one[c]	1.69×10^{-10}	30
	113.096	C7H12O	(e)-2-hepten-1-al	4.39×10^{-11}	21
	115.112	C7H14O	2, 3-dimethylpentanal; trans-2-heptenal; heptanal[c]	3.20×10^{-11}	31, 32
	127.076	C7H10O2	3-methyl-3-hexene-2,5-dione	9.40×10^{-11}	16
	129.127	C8H16O	octanal[f]	3.00×10^{-11}	

	139.112	C9H14O	bicyclo[2.2.1]heptan-2-one,3,3-dimethyl-/sabinaketone/ bicyclo[3.1.1]heptan-2-one, 6,6-dimethyl-[c]	8.85×10^{-12}	33, 34
	123.117<br>141.127	C9H16O	trans-2-nonenal	4.35×10^{-11}	26
	125.132<br>143.143	C9H18O	nonanal	3.60×10^{-11}	35
	153.127	C10H16O	camphor	3.80×10^{-12}	36
	155.154	C10H18O	geraniol; citronellal[c]	1.90×10^{-10}	37, 38
	137.097<br>155.107	C9H14O2	4-methyl-4-octene-1,8-dial (4-MOD)[g]	1.57×10^{-10}	
	139.148<br>157.159	C10H20O	C10 aliphatic carbonyls (decanal)[h]	3.60×10^{-11}	
	151.112<br>169.123	C10H16O2	4-methyl-8-oxo-noennal (4-MON)[g]	1.57×10^{-10}	
	171.175	C11H22O	C11 aliphatic carbonyls (undecanal)[h]	3.60×10^{-11}	
OVOC Carbonyls	177.164<br>195.175	C13H22O	geranyl acetone[g]	1.57×10^{-10}	
		C3H6O	propanal[a]	2.00×10^{-11}	3
	69.034	C4H4O	furan	4.04×10^{-11}	39
	89.060	C4H8O2	acetic acid, ethyl ester; formic acid, 1-methylethyl ester[c]	2.04×10^{-12}	40, 41
	111.044	C6H6O2	aromatic phenol/e,z- and e,e-2,4-hexadienedial/2-furancarbocaldehyde, 5-methyl-/1,2-dihydroxybenzene/1,4-benzenediol/1,3-benzenediol[c]	5.19×10^{-11}	10, 42, 43
OVOC others	113.060	C5H10O2	formic acid, tert-butyl ester/butanoic acid, methyl ester/formic acid, butyl ester/propanoic acid, 2-methyl-, methyl ester/[c]	2.63×10^{-12}	44-49
	115.075	C6H10O2	ethyl crotonate/3-methyl-2,4-pentanedione/2,5-hexanedione/methacrylic acid ethyl ester/acetic acid, propyl ester/acetic acid, 1-methylethyl ester/propanoic acid, ethyl ester[c]	3.25×10^{-11}	50-54

OVOC others	117.091	C6H12O2	butanoic acid, 2-methyl-, methyl ester;methyl valerate;acetic acid, butyl ester;isobutyl acetate;propanoic acid, propyl ester;butanoic acid, ethyl ester[c]	4.17×10^{-11}	44, 45, 48, 55, 56
	129.091	C7H12O2	n-butyl acrylate/4-pentenyl acetate[c]	3.28×10^{-11}	57, 58
	143.107	C8H14O2	1-hydroxy-6-methyl-5-hepten-2-one (OH-6MHO)[i]	6.60×10^{-11}	57
Nitrogen containing	42.033	C2H3N	acetonitrile[b]	2.20×10^{-14}	3
		NH3	ammonia[l]	1.57×10^{-13}	3
	46.029	CH3NO	formamide/formaldoxime[c]	2.97×10^{-12}	63, 59
	70.065	C4H7N	butyronitrile	2.56×10^{-13}	60
	74.024	C2H3NO2	nitroethene	1.20×10^{-12}	61
Sulfur containing	49.011	CH4S	methanethiol	3.30×10^{-11}	3
	63.026	C2H6S	dimethyl sulfide[b]	4.80×10^{-12}	3
	91.057	C4H10S	tert-butylthiol; 2-butanethiol; 1-propanethiol, 2-methyl-; 1-butanethiol; [c]	4.22×10^{-11}	62
	95.016	C2H6O2S	dimethyl sulfone	3.00×10^{-13}	63

NOTES: The compounds calibrated directly by PTR-ToF-MS were m-xylene (m/z 107.086), 1,3,5-trimethylbenzene (m/z 121.101), alpha-pinene (m/z 137.132), methanol (m/z 33.034), acetonitrile (m/z 42.033), acetaldehyde (m/z 45.033), acetone (m/z 59.048), dimethylsulfide (m/z 63.026), methyl vinyl ketone (m/z 71.048), methacrolein (m/z 71.048), methyl ethyl ketone (m/z 73.064), benzene (m/z 79.053), toluene (m/z 93.070). a)measured by GC-MS, b)PTR-MS calibrated, c) k rate averaged from listed isomeric compounds, d) same k_{i+OH} of 4-OPA, e) same k_{i+OH} of 4-hydroxy-2-butenal, f) same k_{i+OH} of hexanal, g) same k_{i+OH} of 6-MHO, h) same k_{i+OH} of nonanal, i) same k_{i+OH} of butyl methacrylate, l) measured by Picarro.

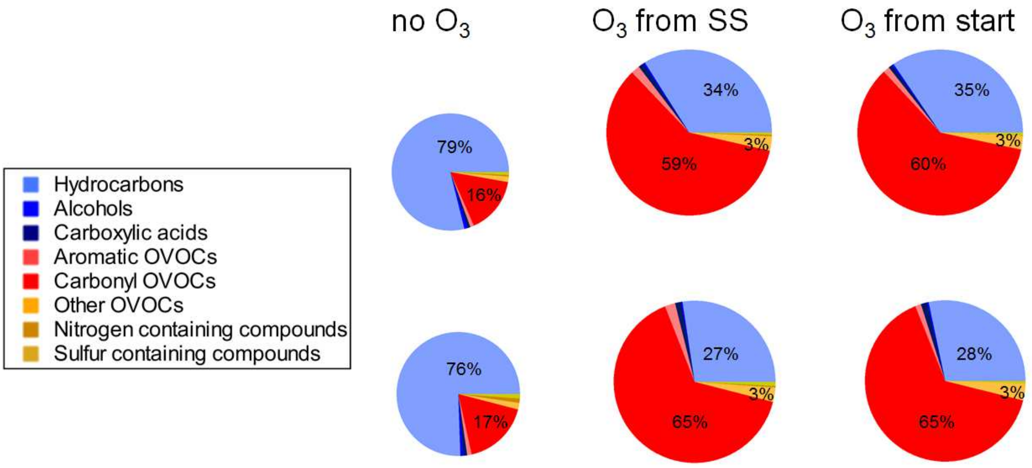

Figure S1. Speciated OH reactivity from occupants wearing long clothing (top row) and short clothing (bottom row), before ozone exposure, with ozone added at the steady state of emissions (afternoon), and with ozone added from the beginning of the experiment. For long clothing, the total OH reactivity represented by each pie chart is 14.2, 32.7, 30.3 s^{-1} for no ozone, ozone from SS, and ozone from start conditions, respectively (top row). For short clothing, it amounts to 15.2, 43.6, 40 s^{-1} for no ozone, ozone from SS, and ozone from start conditions, respectively (bottom row). Steady state values were determined during the last 15 minutes before occupants left the chamber.

Table S3. Ten most reactive volatile organic compounds emitted by four adult volunteers (a, b) wearing long clothing and (c, d) short clothing exposed to ozone (a, c) from start of the experiment and (b, d) in the afternoon, from SS of the emissions. Absolute OH reactivity is reported as mean value ± standard deviation among replicates.

Reactive VOC **(a)**	Absolute OH reactivity [s^{-1}] **(a)**	Reactive VOC **(b)**	Absolute OH reactivity [s^{-1}] **(b)**	Reactive VOC **(c)**	Absolute OH reactivity [s^{-1}] **(c)**	Reactive VOC **(d)**	Absolute OH reactivity [s^{-1}] **(d)**
6-MHO	9.40±2.17	Isoprene	10.3±0.84	6-MHO	14.02±0.57	6-MHO	15.34±0.44
Isoprene	9.29±1.32	6-MHO	9.91±0.02	Isoprene	10.17±0.77	Isoprene	11.17±1.90
4-OPA	1.78±0.40	4-OPA	1.70±0.02	4-OPA	3.16±0.10	4-OPA	2.99±0.12
1,4-butanedial	1.43±0.30	1,4-butanedial	1.3±0.02	1,4-butanedial	1.95±0.03	1,4-butanedial	1.78±0.08
Acetaldehyde	1.41±0.22	Acetaldehyde	1.3±0.01	Acetaldehyde	1.58±0.03	Acetaldehyde	1.61±0.03
Limonene	1.13±0.27	Limonene	0.91±0.01	Limonene	1.00±0.03	Limonene	1.06±0.12

m/z 143.143	1.03±0.10	m/z 143.143	0.74±0.04	Propanal	0.74±0.06	Geranyl acetone	1.02±0.10
Propanal	0.70±0.06	Propanal	0.65±0.09	Geranyl acetone	0.67±0.09	4-methylenehex-5-enal/(3z)-4-methylhexa-3,5-dienal/(3e)-4-methylhexa-3,5-dienal/4-methylcyclohex-3-en-1-one	0.60±0.01
Trans-2-nonenal	0.50±0.04	Geranyl acetone	0.50±0.06	1-hydroxy-6-methyl-5-hepten-2-one (OH-6MHO)	0.70±0.05	m/z 157.159	0.57±0.01
Geranyl acetone	0.45±0.17	1-hydroxy-6-methyl-5-hepten-2-one (OH-6MHO)	0.47±0.02	4-methylenehex-5-enal/(3z)-4-methylhexa-3,5-dienal/(3e)-4-methylhexa-3,5-dienal/4-methylcyclohex-3-en-1-one	0.60±0.04	methyl vinyl ketone (MVK)/methacrolein (MACR)/isoprene hydroxy hydroperoxide (ISOPOOH)	0.55±0.01

Table S4. Calculated O_3 deposition velocity on four occupants (v_{occ}), first-order rate constant for O_3 loss (k_d), measured 6-MHO, 4-OPA, geranyl acetone concentrations, and measured total OH reactivity at SS for each experimental condition. Results indicate mean values ± SD across replicates. $C_{O3,outlet}$ is the chamber outlet O_3 mixing ratio, while $C_{O3,inlet}$ is the chamber inlet O_3 mixing ratio. The air change rate (ACR) was 3.2 h^{-1}, the volume of the chamber (V) was 22.5 m^3 and the area of the reactive surface (A_{occ}) was 7.3 m^2.

clothing/ time O_3 addition	$C_{O3,outlet}$ [ppb]	$C_{O3,inlet}$ [ppb]	v_{occ}[a] [m h^{-1}]	k_{occ} [h^{-1}]	6-MHO [ppb]	4-OPA [ppb]	Geranyl acetone [ppb]	Total OH reactivity[s^{-1}]
long/start	34.8	96.8	17±1.5	5.4±0.4	2.4±0.6	3.6±0.8	0.12±0.04	25±1.5
long/ SS	36.3	105.9	18±0.6	5.8±0.2	2.6±0.01	3.5±0.04	0.13±0.02	29±5
short/start	36.9	100.8	17±0.6	5.5±0.2	3.6±0.2	6.4±0.2	0.2±0.02	31±2.4
short/ SS	35	102.5	19±0.4	6.5±0.1	4±0.11	6.1±0.2	0.3±0.03	35±0.2

[a] assumes O_3 is removed entirely by occupant surfaces.

Table S5. Ozone deposition velocity and OH reactivity of four clean and soiled (worn overnight~8h) t-shirts, and corresponding parameters used.

clothing	$C_{O3,outlet}$ [ppb]	$C_{O3,inlet}$ [ppb]	ACR [h^{-1}]	Volume [m^3]	$A_{clothing}$ [m^2]	$v_{4t\text{-}shirts}$ [m h^{-1}]	$k_{4t\text{-}shirts}$ [h^{-1}]	Total OH reactivity[s^{-1}]
clean	78.34	100	3.2	22.5	2.88	6.9	0.9	< LOD
soiled	72.76	100	3.2	22.5	2.88	9.4	1.2	6.7

Table S6. Ten most reactive volatile organic compounds emitted by (a) four adult volunteers wearing long clothing exposed to ozone, at moderate temperature, low RH (b) at high temperature, low RH, and (c) at high temperature, high RH.

Reactive VOC **(a)**	Absolute OH reactivity [s^{-1}] **(a)**	Reactive VOC **(b)**	Absolute OH reactivity [s^{-1}] **(b)**	Reactive VOC **(c)**	Absolute OH reactivity [s^{-1}] **(c)**
Isoprene	12.61	Isoprene	17.92	Isoprene	12.66
6-MHO	7.69	6-MHO	8.49	6-MHO	10.47
Acetaldehyde	1.86	Acetaldehyde	2.29	4-OPA	1.99
4-OPA	1.31	Propanal	1.32	Acetaldehyde	1.98
1,4-butanedial	1.08	4-OPA	1.32	m/z 143.143	1.85
Limonene	0.92	1,4-butanedial	1.16	1,4-butanedial	1.69
m/z 143.143	0.76	Limonene	1.02	Limonene	1.10
trans-2-nonenal	0.48	m/z 143.143	0.86	m/z 157.159	1.07

m/z 157.159	0.48	Geranyl acetone	0.58	Geranyl acetone	0.92
methyl vinyl ketone (MVK)/ methacrolein (MACR)/ isoprene hydroxy hydroperoxide (ISOPOOH)	0.43	methyl vinyl ketone (MVK)/ methacrolein (MACR)/ isoprene hydroxy hydroperoxide (ISOPOOH)	0.56	Nonanal	0.81

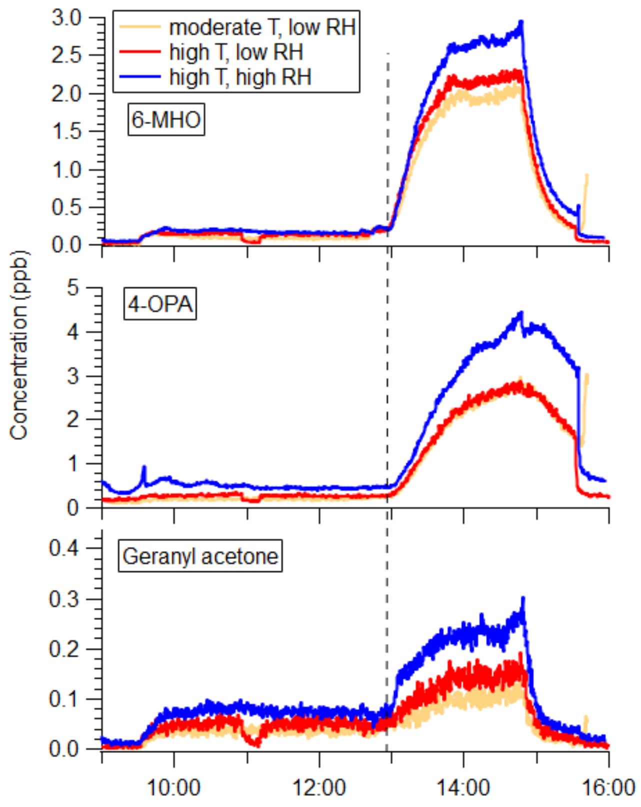

Figure S2. Concentrations of 6-MHO, 4-OPA and geranyl acetone measured in the chamber occupied by four adults from 9:30, wearing long clothing and exposed to moderate/high temperature and low/high relative humidity. The dashed line indicates when ozone was introduced to chamber air. The dip in the experiment at high T and low RH corresponds to measurement of the chamber supply air.

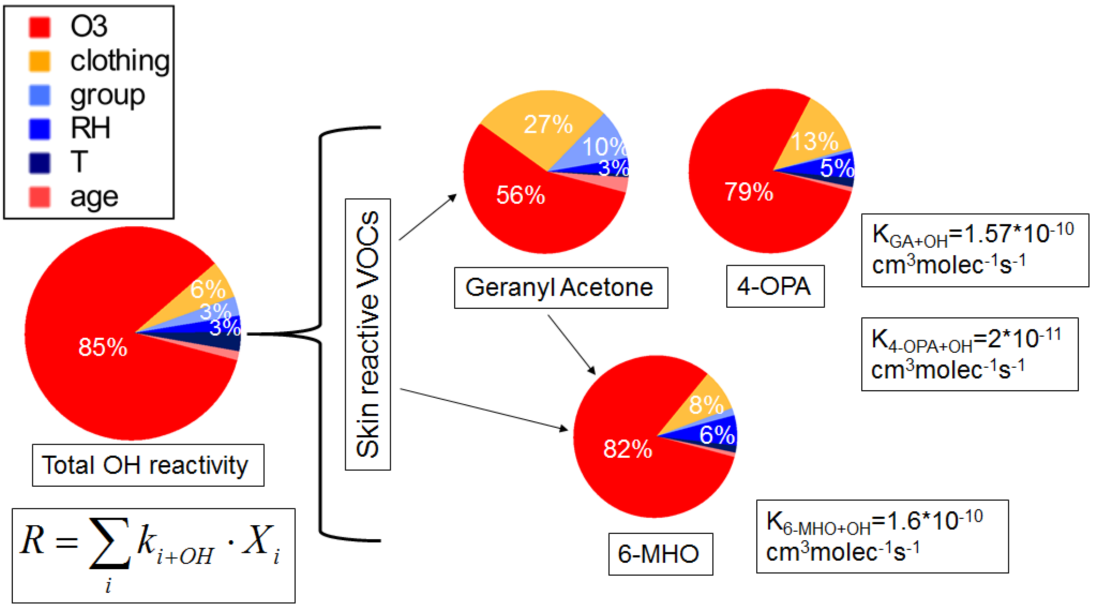

Figure S3. Dominance analysis for the total OH reactivity of human beings, with varying conditions of ozone exposure, short/long clothing, volunteers groups, relative humidity, temperature and age of volunteers (teens/adults/seniors). The pie charts to the right indicate geranyl acetone, 6-MHO, 4-OPA reactivity dominance analyses. Percentage contributions are reported for the main factors. The arrows indicate that geranyl acetone and 6-MHO are the primary products of squalene ozonolysis, while 6-MHO is also generated from geranyl acetone ozonolysis.

References

1. Bekö, G.; Wargocki, P.; Wang, N.; Li, M.; Weschler, C. J.; Morrison, G.; Langer, S.; Ernle, L.; Licina, D.; Yang, S.; Zannoni, N.; Williams, J. The Indoor Chemical Human Emissions and Reactivity (ICHEAR) Project: Overview of Experimental Methodology and Preliminary Results. *Indoor Air* **2020**, *30* (6), 1213–1228. https://doi.org/10.1111/ina.12687.
2. Wang, N.; Zannoni, N.; Ernle, L.; Bekö, G.; Wargocki, P.; Li, M.; Weschler, C. J.; Williams, J. Total OH Reactivity of Emissions from Humans: In Situ Measurement and Budget Analysis. *Environ. Sci. Technol.* **2021**, *55* (1), 149–159. https://doi.org/10.1021/acs.est.0c04206.
3. IUPAC Task Group on Atmospheric Chemical Kinetic Data Evaluation: Datasheets - gas phase: http://iupac.pole-ether.fr/ (iupac preferred values).
4. Atkinson, R.; Arey, J., Atmospheric Degradation of Volatile Organic Compounds. *Chemical Reviews* **2003,** *103*, (12), 4605-4638.
5. Chiorboli, C.; Maldotti, A.; Bignozzian, C.; Carassitik, V. Atmospheric photochemistry: kinetics and mechanism of reactions between aromatic olefins and hydroxyl radical, *Proceedings of the Second European Symposium on Physico-Chemical Behaviour of Atmospheric Pollutants (conference)*, ***1981***, pp 228-233.
6. Atkinson, R.; Baulch, D.; Cox, R.; Crowley, J.; Hampson Jr, R.; Kerr, J.; Rossi, M.; Troe, J., Summary of evaluated kinetic and photochemical data for atmospheric chemistry. *IUPAC Subcommittee on gas kinetic data evaluation for atmospheric chemistry* **2001,** *20*.
7. Teruel, M. A.; Blanco, M. B.; Luque, G. R., Atmospheric fate of acrylic acid and acrylonitrile: rate constants with Cl atoms and OH radicals in the gas phase. *Atmospheric Environment* **2007,** *41*, (27), 5769-5777.

8. Mellouki, A.; Mu, Y., On the atmospheric degradation of pyruvic acid in the gas phase. *Journal of Photochemistry and Photobiology A: Chemistry* **2003,** *157*, (2-3), 295-300.
9. Sørensen, M.; Hurley, M.; Wallington, T.; Dibble, T.; Nielsen, O., Do aerosols act as catalysts in the OH radical initiated atmospheric oxidation of volatile organic compounds? *Atmospheric Environment* **2002,** *36*, (39-40), 5947-5952.
10. Olariu, R. I.; Barnes, I.; Becker, K. H.; Klotz, B., Rate coefficients for the gas-phase reaction of OH radicals with selected dihydroxybenzenes and benzoquinones. *International Journal of Chemical Kinetics* **2000,** *32*, (11), 696-702.
11. Coeur-Tourneur, C.; Henry, F.; Janquin, M.-A.; Brutier, L., Gas-phase reaction of hydroxyl radicals with m-, o- and p-cresol. *International Journal of Chemical Kinetics* **2006,** *38*, (9), 553-562.
12. Coeur-Tourneur, C.; Cassez, A.; Wenger, J. C., Rate Coefficients for the Gas-Phase Reaction of Hydroxyl Radicals with 2-Methoxyphenol (Guaiacol) and Related Compounds. *The Journal of Physical Chemistry A* **2010,** *114*, (43), 11645-11650.
13. Klotz, B.; Barnes, I.; Golding, B. T.; Becker, K.-H., Atmospheric chemistry of toluene-1,2-oxide/2-methyloxepin. *Physical Chemistry Chemical Physics* **2000,** *2*, (2), 227-235.
14. Clifford, G. M.; Wenger, J. C., Rate coefficients for the gas-phase reaction of hydroxyl radicals with the dimethylbenzaldehydes. *International journal of chemical kinetics* **2006,** *38*, (9), 563-569.
15. Atkinson, R.; Arey, J.; Tuazon, E. C.; Aschmann, S. M., Gas-phase reactions of 1, 4-benzodioxan, 2, 3-dihydrobenzofuran, and 2, 3-benzofuran with OH radicals and O3. *International journal of chemical kinetics* **1992,** *24*, (4), 345-358.
16. Aschmann, S. M.; Arey, J.; Atkinson, R., Rate Constants for the Reactions of OH Radicals with 1,2,4,5-Tetramethylbenzene, Pentamethylbenzene, 2,4,5-Trimethylbenzaldehyde, 2,4,5-Trimethylphenol, and 3-Methyl-3-hexene-2,5-dione and Products of OH + 1,2,4,5-Tetramethylbenzene. *The Journal of Physical Chemistry A* **2013,** *117*, (12), 2556-2568.
17. Magneron, I.; Thevenet, R.; Mellouki, A.; Le Bras, G.; Moortgat, G.; Wirtz, K., A study of the photolysis and OH-initiated oxidation of acrolein and trans-crotonaldehyde. *The Journal of Physical Chemistry A* **2002,** *106*, (11), 2526-2537.
18. Mason, S. A.; Arey, J.; Atkinson, R., Kinetics and Products of the OH Radical-Initiated Reaction of 1,4-Butanediol and Rate Constants for the Reactions of OH Radicals with 4-Hydroxybutanal and 3-Hydroxypropanal. *Environmental Science & Technology* **2010,** *44*, (2), 707-713.
19. Fruekilde, P.; Hjorth, J.; Jensen, N. R.; Kotzias, D.; Larsen, B., Ozonolysis at vegetation surfaces: a source of acetone, 4-oxopentanal, 6-methyl-5-hepten-2-one, and geranyl acetone in the troposphere. *Atmospheric Environment* **1998,** *32*, (11), 1893-1902.
20. Bierbach, A.; Barnes, I.; Becker, K. H.; Wiesen, E., Atmospheric chemistry of unsaturated carbonyls: Butenedial, 4-oxo-2-pentenal, 3-hexene-2, 5-dione, maleic anhydride, 3H-furan-2-one, and 5-methyl-3H-furan-2-one. *Environmental science & technology* **1994,** *28*, (4), 715-729.
21. Davis, M.; Gilles, M.; Ravishankara, A.; Burkholder, J. B., Rate coefficients for the reaction of OH with (E)-2-pentenal,(E)-2-hexenal, and (E)-2-heptenal. *Physical Chemistry Chemical Physics* **2007,** *9*, (18), 2240-2248.
22. Baker, J.; Arey, J.; Atkinson, R., Formation and reaction of hydroxycarbonyls from the reaction of OH radicals with 1, 3-butadiene and isoprene. *Environmental science & technology* **2005,** *39*, (11), 4091-4099.

23. Bierbach, A.; Barnes, I.; Becker, K., Product and kinetic study of the OH-initiated gas-phase oxidation of furan, 2-methylfuran and furanaldehydes at≈ 300 K. *Atmospheric Environment* **1995,** *29*, (19), 2651-2660.
24. Renbaum-Wolff, L.; Smith, G. D., "Virtual Injector" Flow Tube Method for Measuring Relative Rates Kinetics of Gas-Phase and Aerosol Species. *The Journal of Physical Chemistry A* **2012,** *116*, (25), 6664-6674.
25. Grosjean, D.; Williams II, E. L., Environmental persistence of organic compounds estimated from structure-reactivity and linear free-energy relationships. Unsaturated aliphatics. *Atmospheric Environment. Part A. General Topics* **1992,** *26*, (8), 1395-1405.
26. Gao, T.; Andino, J. M.; Rivera, C. C.; Márquez, M. F., Rate constants of the gas-phase reactions of OH radicals with trans-2-hexenal, trans-2-octenal, and trans-2-nonenal. *International Journal of Chemical Kinetics* **2009,** *41*, (7), 483-489.
27. Xing, J.-H.; Ono, M.; Kuroda, A.; Obi, K.; Sato, K.; Imamura, T., Kinetic study of the daytime atmospheric fate of (Z)-3-hexenal. *The Journal of Physical Chemistry A* **2012,** *116*, (33), 8523-8529.
28. Aschmann, S. M.; Arey, J.; Atkinson, R., Atmospheric chemistry of selected hydroxycarbonyls. *The Journal of Physical Chemistry A* **2000,** *104*, (17), 3998-4003.
29. Smith, A. M.; Rigler, E.; Kwok, E. S. C.; Atkinson, R., Kinetics and Products of the Gas-Phase Reactions of 6-Methyl-5-hepten-2-one and trans-Cinnamaldehyde with OH and NO3 Radicals and O3 at 296 ± 2 K. *Environmental Science & Technology* **1996,** *30*, (5), 1781-1785.
30. Baker, J.; Arey, J.; Atkinson, R., Kinetics of the Gas-Phase Reactions of OH Radicals, NO3 Radicals and O3 with Three C7-Carbonyls Formed From The Atmospheric Reactions of Myrcene, Ocimene and Terpinolene. *Journal of Atmospheric Chemistry* **2004,** *48*, (3), 241-260.
31. Albaladejo, J.; Ballesteros, B.; Jiménez, E.; Martín, P.; Martínez, E., A PLP–LIF kinetic study of the atmospheric reactivity of a series of C4–C7 saturated and unsaturated aliphatic aldehydes with OH. *Atmospheric environment* **2002,** *36*, (20), 3231-3239.
32. Tuazon, E. C.; Aschmann, S. M.; Nguyen, M. V.; Atkinson, R., H-atom abstraction from selected C · H bonds in 2, 3-dimethylpentanal, 1, 4-cyclohexadiene, and 1, 3, 5-cycloheptatriene. *International Journal of Chemical Kinetics* **2003,** *35*, (9), 415-426.
33. Atkinson, R.; Aschmann, S. M., Atmospheric chemistry of the monoterpene reaction products nopinone, camphenilone, and 4-acetyl-1-methylcyclohexene. *Journal of atmospheric chemistry* **1993,** *16*, (4), 337-348.
34. Carrasco, N.; Picquet-Varrault, B.; Doussin, J.-F., Kinetic and product study of the gas-phase reaction of sabinaketone with OH radical. *International Journal of Chemical Kinetics* **2007,** *39*, (7), 415-421.
35. Bowman, J. H.; Barket, D. J.; Shepson, P. B., Atmospheric chemistry of nonanal. *Environmental science & technology* **2003,** *37*, (10), 2218-2225.
36. Ceacero-Vega, A. A.; Ballesteros, B.; Bejan, I.; Barnes, I.; Jiménez, E.; Albaladejo, J., Kinetics and mechanisms of the tropospheric reactions of menthol, borneol, fenchol, camphor, and fenchone with hydroxyl radicals (OH) and chlorine atoms (Cl). *The Journal of Physical Chemistry A* **2012,** *116*, (16), 4097-4107.
37. Forester, C. D.; Ham, J. E.; Wells, J., Geraniol (2, 6-dimethyl-2, 6-octadien-8-ol) reactions with ozone and OH radical: Rate constants and gas-phase products. *Atmospheric Environment* **2007,** *41*, (6), 1188-1199.
38. Harrison, J.; Ham, J.; Wells, J., Citronellal reactions with ozone and OH radical: Rate constants and gas-phase products detected using PFBHA derivatization. *Atmospheric Environment* **2007,** *41*, (21), 4482-4491.

39. Atkinson, R., Kinetics and mechanisms of the gas-phase reactions of the hydroxyl radical with organic compounds under atmospheric conditions. *Chemical Reviews* **1986,** *86*, (1), 69-201.
40. Picquet, B.; Heroux, S.; Chebbi, A.; Doussin, J. F.; Durand-Jolibois, R.; Monod, A.; Loirat, H.; Carlier, P., Kinetics of the reactions of OH radicals with some oxygenated volatile organic compounds under simulated atmospheric conditions. *International journal of chemical kinetics* **1998,** *30*, (11), 839-847.
41. Pimentel, A. S.; Tyndall, G. S.; Orlando, J. J.; Hurley, M. D.; Wallington, T. J.; Sulbaek Andersen, M. P.; Marshall, P.; Dibble, T. S., Atmospheric chemistry of isopropyl formate and tert-butyl formate. *International Journal of Chemical Kinetics* **2010,** *42*, (8), 479-498.
42. Bierbach, A.; Barnes, I.; Becker, K., Rate coefficients for the gas-phase reactions of hydroxyl radicals with furan, 2-methylfuran, 2-ethylfuran and 2, 5-dimethylfuran at 300±2 K. *Atmospheric Environment. Part A. General Topics* **1992,** *26*, (5), 813-817.
43. Klotz, B.; Barnes, I.; Becker, K.-H., Kinetic study of the gas-phase photolysis and OH radical reaction of E,Z- and E,E-2,4-Hexadienedial. *International Journal of Chemical Kinetics* **1999,** *31*, (10), 689-697.
44. Ferrari, C.; Roche, A.; Jacob, V.; Foster, P.; Baussand, P., Kinetics of the reaction of OH radicals with a series of esters under simulated conditions at 295 K. *International Journal of Chemical Kinetics* **1996,** *28*, (8), 609-614.
45. Cometto, P. M.; Daële, V.; Idir, M.; Lane, S. I.; Mellouki, A., Reaction Rate Coefficients of OH Radicals and Cl Atoms with Ethyl Propanoate, n-Propyl Propanoate, Methyl 2-Methylpropanoate, and Ethyl n-Butanoate. *The Journal of Physical Chemistry A* **2009,** *113*, (40), 10745-10752.
46. Le Calve, S.; Le Bras, G.; Mellouki, A., Temperature dependence for the rate coefficients of the reactions of the OH radical with a series of formates. *The Journal of Physical Chemistry A* **1997,** *101*, (30), 5489-5493.
47. Szilágyi, I.; Dóbé, S.; Bérces, T.; Márta, F.; Viskolcz, B., Direct kinetic study of reactions of hydroxyl radicals with alkyl formates. *Zeitschrift für Physikalische Chemie* **2004,** *218*, (4), 479-492.
48. Schütze, N.; Zhong, X.; Kirschbaum, S.; Bejan, I.; Barnes, I.; Benter, T., Relative kinetic measurements of rate coefficients for the gas-phase reactions of Cl atoms and OH radicals with a series of methyl alkyl esters. *Atmospheric Environment* **2010,** *44*, (40), 5407-5414.
49. Blanco, M. B.; Taccone, R. A.; Lane, S. I.; Teruel, M. A., On the OH-initiated degradation of methacrylates in the troposphere: Gas-phase kinetics and formation of pyruvates. *Chemical physics letters* **2006,** *429*, (4-6), 389-394.
50. Dagaut, P.; Wallington, T. J.; Liu, R.; Kurylo, M. J., A kinetic investigation of the gas-phase reactions of hydroxyl radicals with cyclic ketones and diones: mechanistic insights. *The Journal of Physical Chemistry* **1988,** *92*, (15), 4375-4377.
51. Holloway, A.-L.; Treacy, J.; Sidebottom, H.; Mellouki, A.; Daële, V.; Le Bras, G.; Barnes, I., Rate coefficients for the reactions of OH radicals with the keto/enol tautomers of 2, 4-pentanedione and 3-methyl-2, 4-pentanedione, allyl alcohol and methyl vinyl ketone using the enols and methyl nitrite as photolytic sources of OH. *Journal of Photochemistry and Photobiology A: Chemistry* **2005,** *176*, (1-3), 183-190.
52. Teruel, M. A.; Benitez-Villalba, J.; Caballero, N.; Blanco, M. B., Gas-Phase oxidation of methyl crotonate and ethyl crotonate. kinetic study of their reactions toward OH radicals and Cl atoms. *The Journal of Physical Chemistry A* **2012,** *116*, (24), 6127-6133.

53. Le Calvé, S.; Le Bras, G.; Mellouki, A., Kinetic studies of OH reactions with Iso-propyl, Iso-butyl, Sec-butyl, and Tert-butyl acetate. *International journal of chemical kinetics* **1997,** *29*, (9), 683-688.
54. Veillerot, M.; Foster, P.; Guillermo, R.; Galloo, J. C., Gas-phase reaction of n-butyl acetate with the hydroxyl radical under simulated tropospheric conditions: Relative rate constant and product study. *International Journal of Chemical Kinetics* **1996,** *28*, (4), 235-243.
55. Stemmler, K.; Mengon, W.; Alistair Kerr, J., Hydroxyl-radical-initiated oxidation of isobutyl isopropyl ether under laboratory conditions related to the troposphere Product studies and proposed mechanism. *Journal of the Chemical Society, Faraday Transactions* **1997,** *93*, (16), 2865-2875.
56. Blanco, M. B.; Bejan, I.; Barnes, I.; Wiesen, P.; Teruel, M. A., OH-Initiated Degradation of Unsaturated Esters in the Atmosphere: Kinetics in the Temperature Range of 287−313 K. *The Journal of Physical Chemistry A* **2009,** *113*, (20), 5958-5965.
57. Williams, D. C.; O'Rji, L. N.; Stone, D. A., Kinetics of the reactions of OH radicals with selected acetates and other esters under simulated atmospheric conditions. *International Journal of Chemical Kinetics* **1993,** *25*, (7), 539-548.
58. Nizamov, B.; Dagdigian, P. J., Spectroscopic and kinetic investigation of methylene amidogen by cavity ring-down spectroscopy. *The Journal of Physical Chemistry A* **2003,** *107*, (13), 2256-2263.
59. Borduas, N.; da Silva, G.; Murphy, J. G.; Abbatt, J. P. D., Experimental and Theoretical Understanding of the Gas Phase Oxidation of Atmospheric Amides with OH Radicals: Kinetics, Products, and Mechanisms. *The Journal of Physical Chemistry A* **2015,** *119*, (19), 4298-4308.
60. Sun, J.; Tang, Y.; Sun, H.; Pan, Y.; Jia, X.; Pan, X.; Wang, R., Mechanistic and kinetic study of the OH+ C2H5CN reaction. *Chemical Physics Letters* **2008,** *463*, (4-6), 315-321.
61. Nielsen, O. J.; Jørgensen, O.; Donlon, M.; Sidebottom, H. W.; O'Farrell, D. J.; Treacy, J., Rate constants for the gas-phase reactions of OH radicals with nitroethene, 3-nitropropene and 1-nitrocyclohexene at 298 K and 1 atm. *Chemical physics letters* **1990,** *168*, (3-4), 319-323.
62. Barnes, I.; Bastian, V.; Becker, K.; Fink, E.; Nelsen, W., Oxidation of sulphur compounds in the atmosphere: I. Rate constants of OH radical reactions with sulphur dioxide, hydrogen sulphide, aliphatic thiols and thiophenol. *Journal of atmospheric chemistry* **1986,** *4*, (4), 445-466.
63. Falbe-Hansen, H.; Sørensen, S.; Jensen, N.; Pedersen, T.; Hjorth, J., Atmospheric gas-phase reactions of dimethylsulphoxide and dimethylsulphone with OH and NO3 radicals, Cl atoms and ozone. *Atmospheric Environment* **2000,** *34*, (10), 1543-1551.
